# Formation of phenotypic lineages in *Salmonella enterica* by a pleiotropic fimbrial switch

**DOI:** 10.1371/journal.pgen.1007677

**Published:** 2018-09-25

**Authors:** Lucía García-Pastor, María Antonia Sánchez-Romero, Gabriel Gutiérrez, Elena Puerta-Fernández, Josep Casadesús

**Affiliations:** 1 Departamento de Genética, Facultad de Biología, Universidad de Sevilla, Sevilla, Spain; 2 Instituto de Recursos Naturales y Agrobiología de Sevilla (IRNAS, CSIC), Sevilla, Spain; Baylor College of Medicine, UNITED STATES

## Abstract

The *std* locus of *Salmonella enterica*, an operon acquired by horizontal transfer, encodes fimbriae that permit adhesion to epithelial cells in the large intestine. Expression of the *std* operon is bistable, yielding a major subpopulation of Std^OFF^ cells (99.7%) and a minor subpopulation of Std^ON^ cells (0.3%). In addition to fimbrial proteins, the *std* operon encodes two proteins, StdE and StdF, that have DNA binding capacity and control transcription of loci involved in flagellar synthesis, chemotaxis, virulence, conjugal transfer, biofilm formation, and other cellular functions. As a consequence of StdEF pleiotropic transcriptional control, Std^ON^ and Std^OFF^ subpopulations may differ not only in the presence or absence of Std fimbriae but also in additional phenotypic traits. Separation of Std^OFF^ and Std^ON^ lineages by cell sorting confirms the occurrence of lineage-specific features. Formation of Std^OFF^ and Std^ON^ lineages may thus be viewed as a rudimentary bacterial differentiation program.

## Author summary

We show that the *std* fimbrial operon of *Salmonella enterica* undergoes bistable expression, a trait far from exceptional among loci that encode components of the bacterial envelope. However, an unsuspected trait of the *std* operon is the presence of two genes that encode pleiotropic regulators of gene expression. Indeed, StdE and StdF are DNA-binding proteins that control transcription of hundreds of genes. As a consequence, StdEF govern multiple phenotypic traits, and the fimbriated and non-fimbriated *Salmonella* lineages may differ in motility, virulence, conjugal transfer, biofilm formation, and potentially in other phenotypic features. We hypothesize that pleiotropic control of gene expression by StdEF may contribute to adapt the non-fimbriated lineage to acute infection and the fimbriated lineage to chronic infection.

## Introduction

Phenotypic differences in isogenic bacterial cells grown in the same environment can be a consequence of noisy gene expression [1,2]. In other cases, the occurrence of distinct phenotypes is a programmed event that causes bistability, the split of the bacterial population into two lineages [3–5]. The mechanisms that cause bistability include genetic rearrangement, expansion and contraction of DNA sequence repeats, epigenetic control of gene expression by DNA methylation, and formation of regulatory feedback loops transmissible to daughter cells [5]. This variety, obviously indicative of independent evolution, may suggest that the ability of a given bacterial species to diversify into phenotypic lineages is a product of natural selection. Indeed, game theory shows that lineage formation can have selective value either as a division of labour or as a bet hedging [3,6–8]. In both kinds of strategies, the key biological entity is not the individual cell but the population [9]. Division of labour permits use of resources that are not available to a single cell type. In bet hedging, the fitness of each cell type is higher under different circumstances, and differentiation into distinct lineages preadapts the population to environmental changes.

In bacterial pathogens, phenotypic variation is often associated with virulence [10]. Variation in surface antigens such as flagellin, fimbrial and non-fimbrial adhesins, and the lipopolysaccharide help to evade the immune system [11]. In *Salmonella*, bistability in surface structures can also prevent cross-immunity between different serotypes [12]. Additional benefits from the formation of bacterial lineages include protection against host defence mechanisms other than the immune system [13], resistance to bacteriophages [14] and antimicrobial substances [15,16], and optimization of metabolic adaptation [17].

Fimbriae are virulence factors that promote attachment of bacterial cells to specific host tissues [18]. In *S*. *enterica* serotype Typhimurium, the ability of fimbriae to agglutinate yeast or red blood cells was described in 1966 [19]. Later studies have identified a large number of fimbrial loci including *fim* [20], *csg* [21], [22], *stf* [23,24], *saf* [25], *stb*, *stc*, *std*, *sth*, *sti*, and *stj* [26].

This study deals with *std*, one of the *Salmonella* operons initially identified by genome sequencing [26]. Absence of *std* in enterobacterial genera other than *Salmonella* suggests acquisition by horizontal transfer [27]. Std fimbriae bind specific receptors of the cecal mucose in the large intestine, and may play a role in chronic intestinal infection [28,29]. The *std* operon contains six genes (*stdABCDEF*) which are co-transcribed from a promoter located upstream of *stdA* [30]. Expression of the *std* operon is under transcriptional control by DNA adenine methylation and by HdfR, a poorly known LysR-like transcription factor [31].

This study shows that *std* expression is bistable, a trait shared with other adhesin-encoding operons [32]. A difference, however, is that *std* appears to be more than just a fimbrial operon: two products of the *std* operon, StdE and StdF, have DNA-binding capacity and activate or repress transcription of multiple genes. As a consequence, Std^ON^ and Std^OFF^ subpopulations may differ not only in the possession of Std fimbriae but in additional phenotypic traits. Pleiotropic control of gene expression upon formation of Std^ON^ and Std^OFF^ lineages may thus be viewed as an example of rudimentary, inconspicuous bacterial cell differentiation.

## Results

### Bistable expression of the *std* operon

**Fig 1:**
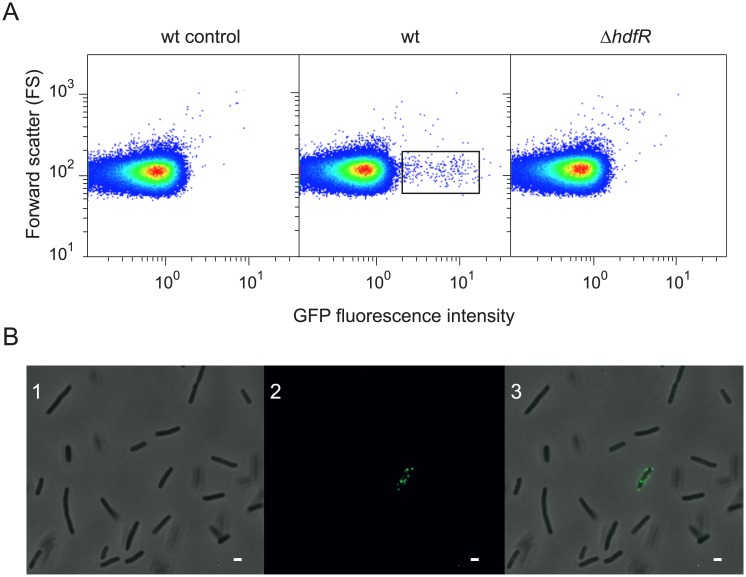
Bistable expression of the *std* operon. **A.** Single cell analysis of GFP fluorescence intensity by flow cytometry in a strain carrying an *stdA*::*gfp* fusion in different genetic backgrounds (wt and Δ*hdfR*). On the left, a negative control is shown (*S*. *enterica* cells without *gfp* fusion). **B.** Detection of Std fimbriae by immunofluorescence microscopy. Panel 1 shows a phase-contrast image of *S*. *enterica* cells. Panel 2 shows detection of Std fimbriae with anti-StdA antiserum and goat anti-rabbit antibody conjugated to FITC (green signal). In panel 3, both channels are merged. Scale bar: 1 μm.

Single cell analysis of *std* expression was performed by flow cytometry in a *stdA*::*gfp* strain (SV9597). This strain carries a *gfp* transcriptional fusion downstream of *stdA*. Insertion of *gfp* does not cause polar effects on downstream genes of the *std* operon. A representative experiment presented in Fig 1, panel A reveals the existence of two subpopulations: a major subpopulation that does not show *stdA*::*gfp* expression (Std^OFF^, >99% of cells) and a minor Std^ON^ subpopulation that shows *stdA*::*gfp* expression (Std^ON^, <1% of cells). Formation of the Std^ON^ subpopulation was abolished in a strain that lacks HdfR, a LysR-type transcription factor previously described as an activator of *std* transcription [31] (Fig 1, panel A). HdfR is thus necessary for formation of the Std^ON^ lineage.

Evidence that expression of the *std* operon occurs only in a subpopulation of *S*. *enterica* cells was confirmed by fluorescence microscopy. Wild type *S*. *enterica* cells were labelled with rabbit anti-StdA serum and goat anti-rabbit IgG-FITC conjugated antibody. A small fraction of cells harboured Std fimbriae, thereby confirming the existence of Std^OFF^ and Std^ON^ subpopulations (Fig 1, panel B).

### Genome-wide consequences of *std* expression: Transcriptomic analysis

Evidence that the products of two downstream genes of the *std* operon, StdE and StdF, controlled the expression of genes located outside the *std* operon [30] led us to investigate the extent of gene regulation by StdE and StdF. Transcriptomic analysis was performed in a strain that constitutively expressed *stdEF* (StdEF^+^, SV8141) and in a strain carrying an in-frame deletion of both genes (StdEF^–^, SV8142). In strain StdEF^+^, both *stdE* and *stdF* are transcribed from the P_L*tetO*_ promoter inserted upstream of *stdE* on the *Salmonella* chromosome, and the native *stdA* promoter and all genes upstream of *stdE* are deleted [30] (S1 Fig). Choice of P_L*tetO*_ was based on the fact that this promoter renders moderate, constitutive expression [33,34]. Strain StdEF^−^contained the same insertion of the P_L*tetO*_ promoter and a complete deletion of the *std* operon (S1 Fig, panel B). Transcriptomic analysis was performed using a previously described *S*. *enterica* ser. Typhimurium SL1344 gene array [35]. Raw data from transcriptomic analysis were deposited at the Gene Expression Omnibus (GEO) database (http://www.ncbi.nlm.nih.gov/geo/), with accession number GSE45488.

A large number of *S*. *enterica* genes showed different RNA levels in StdEF^+^ and StdEF^−^strains. Table 1 include only loci whose RNA levels differed more than 4-fold between the StdEF^+^ and StdEF^−^strains. A detailed gene description of these loci, including each individual fold change, is provided in S1 Table.

**Table 1:** Genes regulated by StdE and/or StdF.

	GENE/CLUSTER	FUNCTION
**DOWNREGULATED**	*avrA*, *sprB*, *hilC*, *orgCBA*, *prgKJIH*, *hilD*, *hilA*, *iagB*, *sicP*, *iacP*, *sipADCB*, *sicA*, *spaSROPQ*, *invJICBAEGFH*	Pathogenicity island 1 (SPI-1)
	*siiABCDEF*	Pathogenicity island 4 (SPI-4)
	*pipB*, *pipC*, *sopB*	Pathogenicity island 5 (SPI-5)
	*ygiD*	Biofilm formation
	*flgNMACDEFGHIJKL*, *flhEAB*, *motAB*, *flhDC*, *fliZABCDSTEFGHIJKLMNO*, *fljB*	Motility
	*cheZYBRMWA*, *aer*, *tsr*, *trg*, *tcp*	Chemotaxis
	*sopA*, *sopE2*, *sopD*, *sopE*, *slrp*, *sptP*, *steB*	SPI-1 & SPI-2 Effectors^a^
	*modABC*, *cadBA*, *iroBC*,	Metabolism
	*srfABC*, *rtsAB*, *asrAB*, *tdcE*, *yhhP*, *yhjH*, *ydcX*, *ycgR*, *yghW*, SL1177, *sdiA*	Miscellaneous
	*yeaQ*, *ymdA*, SL1028, SL1236, SL1235, SL1263, SL1867, SL1896, SL2283, SL3112, SL3126, SL3128-SL3130, SL3189, SL3569,SL4247-SL4249	Unknown
	Total number: 143	
**UPREGULATED**	*traYALEP*, SLP2_0079	Conjugation
	*fhuA*, *ygiK*, *sitA*	Metabolism
	SL0982, *nlpC*, *ordL*, *bglJ*, *hdfR*, SL1062, *entF*	Miscellaneous
	SL0502, SL3096, SL3097, SL3099, SL3100, SL3101, SL3103, SL3143, SL3144, SL3653, SL4480	Unknown
	Total number: 27	

^a^ Encoded outside SPI-1 and SPI-2

Downregulation by StdEF was observed at many loci, suggesting that StdE and StdF are often repressors of gene expression in *Salmonella*. The list of downregulated loci was heterogeneous, and included genes located in pathogenicity islands SPI-1, SPI-4 and SPI-5, the *flhDC* master regulatory operon, the flagellar operons *flg*, *flh*, *fli*, *fljB* and *motAB*, the *che* operon, the *trg*, *tcp*, *tsr* and *aer genes* involved in chemotaxis, the poorly known *ygiD* gene involved in biofilm formation, and additional loci involved in metabolism or having miscellaneous or unknown functions.

The list of upregulated loci was also heterogeneous, and included the *tra* operon encoded on the pSLT plasmid, genes involved in metabolism, and loci with miscellaneous or unknown functions. An interesting observation was the presence of *hdfR* among the StdEF-upregulated genes. Because the HdfR gene product is a transcriptional activator of *std* expression [31] (Fig 1A), upregulation of *hdfR* transcription by StdE and StdF may suggest the existence of a positive feedback loop for autogenous regulation of the *std* operon.

**Fig 2:**
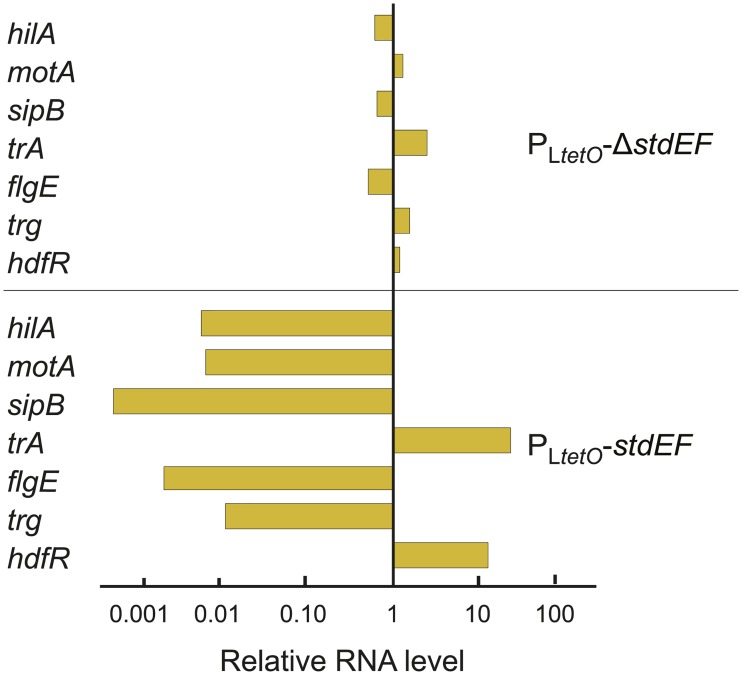
Validation of microarray data. RNA levels of some of the genes under StdEF control identified by microarray analysis in strains P_L*tetO*_-*stdEF* (SV8141) and P_L*tetO*_-Δ*stdEF* (SV8142). For each locus, data are normalized to the RNA level obtained in the wild type (which was set to "1" in all cases). The RNA levels were determined in ≥ 3 biological replicates, and a representative experiment is shown.

Validation of transcriptomic data was achieved by quantitative real time PCR, monitoring RNA production at loci that had shown differential expression in StdEF^+^ and StdEF^−^strains. The list includes genes involved in motility (*motA*, *flgE*), conjugation (*traA*), chemotaxis (*trg*), virulence (*hilA*, *sipB*) and transcriptional regulation (*hdfR*). All the loci analyzed were found to be under StdEF control (Fig 2), and the gene expression changes detected by RT-PCR correlated well with those obtained by transcriptomic analysis.

### Subcellular localization of StdE and StdF

**Fig 3:**
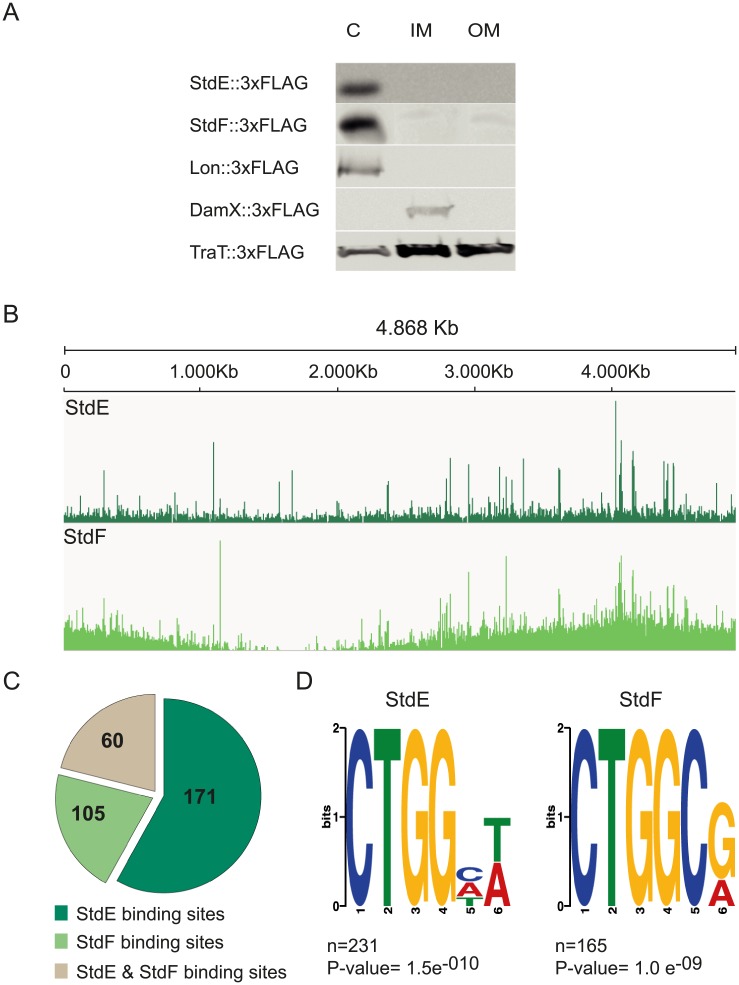
Genome-wide identification of StdE and StdF binding sites by ChIP-seq. **A.** Subcellular localization of StdE and StdF proteins in cellular fractions (cytoplasm (c), inner membrane (IM) and outer membrane (OM)) using 3xFLAG-tagged versions of the proteins (SV9324 and SV9325, respectively). Controls used for each fraction are Lon, DamX and TraT. **B.** Visualization of StdE and StdF ChIP-seq data using the Integrative Genomics Viewer (IGV) for *S*. Typhimurium SL 1344. StdE represents the difference between the StdE IP sample and the mock IP control and the same is valid for StdF. **C.** The pie chart shows the number of StdE, StdF and StdE & StdF binding sites along the *Salmonella enterica* genome. **D.** StdE and StdF binding motifs identified by Meme.

The subcellular localization of StdE and StdF was determined using chromosomal 3xFLAG-tagged versions of the StdE and StdF proteins (strains SV9324 and SV9325, respectively). Since the *std* operon is only expressed in a small fraction of cells in the wild type (Fig 1), this analysis was performed in a Dam^−^ background [31]. Electrophoretic separation of cell fractions (cytoplasm, cytoplasmic membrane and outer membrane) was followed by Western blot analysis of the separated cell samples using a commercial anti-FLAG antibody. DamX, TraT and Lon were used as localization controls [36]. Both StdE and StdF were found in the cytoplasmic fraction (Fig 3, panel A).

### ChIP-seq analysis of StdE and StdF DNA binding ability

Cytoplasmic localization of StdE and StdF, together with the evidence that they control the expression of multiple *S*. *enterica* genes, raised the possibility that these proteins might have DNA binding ability. This hypothesis was tested by chromatin immunoprecipitation followed by DNA sequencing (ChIP-seq). The strain used for ChIP-seq carried the *stdEF* genes under the control of P_L*tetO*_ (as in the strain used in transcriptomic analysis), and contained a StdF variant labeled at its C-terminus with a 3xFLAG epitope (P_L*tetO*_*-stdEF-*3xFLAG; SV7850). This construct allowed the detection of StdE with a cognate anti-StdE antibody while StdF was detected with an anti-FLAG antibody.

Raw and processed data from ChIP-seq analysis have been deposited at the Gene Expression Omnibus (GEO) database (http://www.ncbi.nlm.nih.gov/geo/), with accession number GSE113562.

ChIP-seq analysis revealed that StdE and StdF bind multiple sites in the *S*. *enterica* genome (Fig 3, panel B). The number of DNA sequence reads detected upon immunoprecipitation with the StdE antibody was higher than that found for StdF (Fig 3, panel B), suggesting that StdE and StdF may bind DNA independently, and that StdE may bind more efficiently than StdF. The pie chart shown in Fig 3, panel C, summarizes the number of binding sites detected for StdE (171), for StdF (105), and for both StdE and StdF (60). Peak boundary sequences for StdE (231) and StdF (165) were extracted from the reference genome, and were analyzed with a motif-finding algorithm. Distinct 6-bp motifs for StdE and StdF binding were identified (Fig 3D).

Binding of StdE and/or StdF to specific promoters or upstream regulatory regions permitted a tentative interpretation of data from transcriptomic analysis. For instance, binding of StdEF was detected upstream of the *flhDC* flagellar operon and also upstream of the conjugal transfer *tra* operon (see below). StdE and StdF binding sites were also detected within coding regions, perhaps indicating the existence of uncharacterized promoters [37]. On the other hand, intra-ORF binding has been documented previously for other transcriptional regulators [38–40].

### Genome-wide consequences of *std* expression: Phenotypic and genetic analysis

Phenotypic validation of the observations provided by gene expression analysis and ChIP-seq was pursued by monitoring motility, epithelial cell invasion, biofilm formation, and conjugal transfer of the virulence plasmid. In certain cases, genetic analysis was also performed to identify regulatory mechanisms and epistatic relationships. Relevant observations were as follows:

#### Flagella and chemotaxis

**Fig 4:**
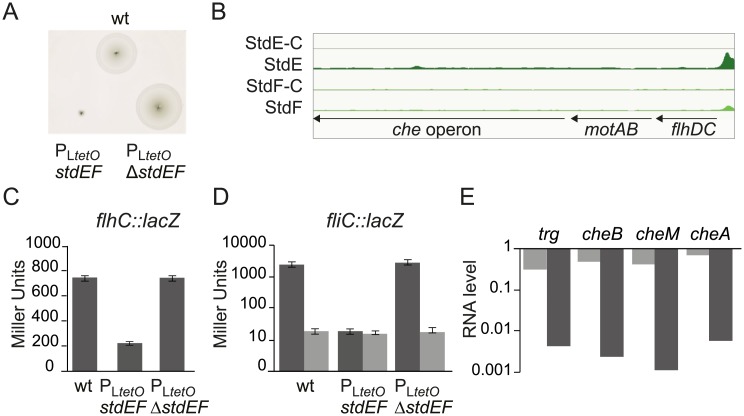
Flagellar control by StdE and StdF. **A.** Motility of strains SL1344 (wt), P_L*tetO*_-*stdEF* (SV8141) and P_L*tetO*_-Δ*stdEF* (SV8142). The experiments were performed for at least three biological replicates, and a representative experiment is shown. **B.** StdE and StdF binding peaks in the *flhDC* region. StdE-C and StdF-C indicate binding peaks found for the mock IP-sample. **C.** β-galactosidase activity of a *flhC*::*lac*Z fusion. Constitutive expression of *stdEF* (SV9289) results in low level of *flhC* expression, compared with the wild type strain (SV9288) and with the control strain P_L*tetO*_-Δ*stdEF* (SV9290). **D**. β-galactosidase activity of a *fliC*::*lacZ* fusion showing that StdE and StdF regulation of the flagellar system take place through FlhDC. Dark grey bars represent FlhC^+^ and light grey bars represent FlhC^–^. **E**. Ratios (P_L*tetO*_-*stdEF* / P_L*tetO*_-Δ*stdEF)* of mRNA levels of genes involved in chemotaxis in the strains P_L*tetO*_-*stdEF* and P_L*tetO*_-Δ*stdEF*. RNA levels were determined for at least 3 biological replicates and a representative experiment is shown. As above, dark grey bars represent FlhC^+^ and light grey bars represent FlhC^–^.

Strain P_L*tetO*_-*stdEF* (SV8141) showed reduced motility on soft agar (Fig 4, panel A), indicating that expression of StdE and StdF reduces motility.

StdE and StdF binding was detected upstream of *flhDC* (Fig 4, panel B), the master operon for flagellar regulation [41]. This observation led us to test whether *flhDC* transcription was StdEF-dependent, and whether StdEF-mediated regulation of downstream loci in the flagellar and chemotaxis gene networks depended or not on FlhDC. Conclusions from these experiments can be summarized as follows:

1. An *flhC*::*lacZ* fusion was found to be repressed by StdEF (Fig 4, panel C), thereby providing a tentative explanation for downregulation of motility.
2. A *fliC*::*lacZ* fusion was repressed by StdEF in the presence of FlhDC only (Fig 4, panel D), suggesting that StdEF-mediated repression of *flhDC* transcription may be transmitted to downstream genes in the flagellar regulon.
3. Quantitative RT-PCR in FlhC^+^ and FlhC^−^backgrounds revealed that StdEF-mediated repression of *cheA*, *cheM*, *cheB*, and *trg* occurred in an FlhDC^+^ background only (Fig 4, panel E), suggesting that downregulation of chemotaxis genes by StdEF is mediated by repression of the flagellar master operon *flhDC*. This tentative conclusion is consistent with the absence of StdE and StdF binding sites in chemotaxis operons (Fig 4, panel B), and with the fact that regulation of chemotaxis is known to be dependent on the FlhDC master regulator [42].

#### *Salmonella* pathogenicity island 1

**Fig 5:**
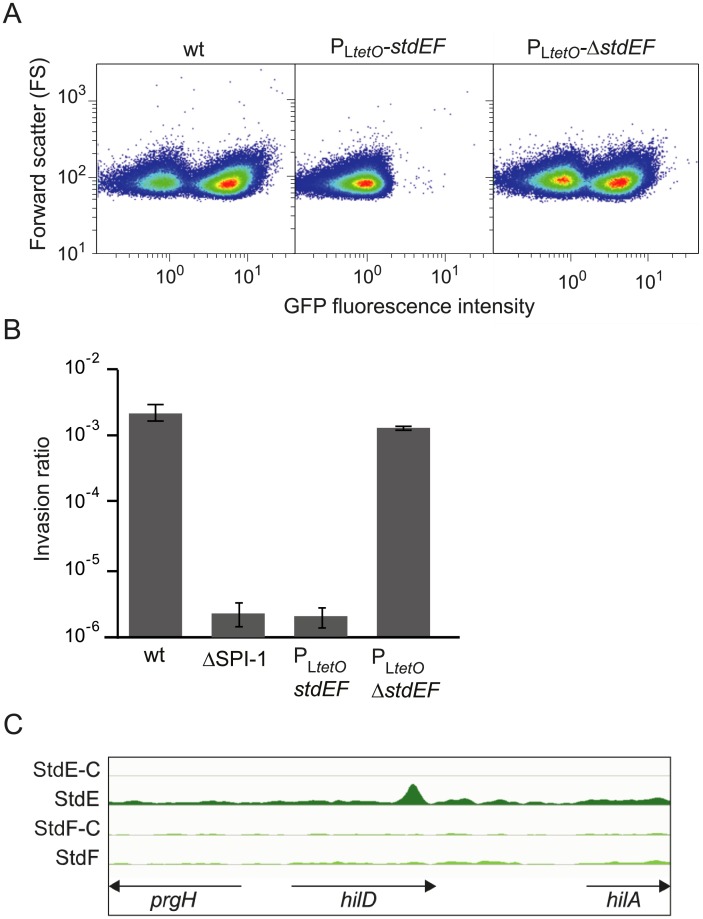
SPI-1 control by StdE. **A.** GFP fluorescence intensity in strains *sipB*::*gfp* (SV7884), *sipB*::*gfp* P_L*tetO*_-*stdEF* (SV7885), and *sipB*::*gfp* P_L*tetO*_-Δ*stdEF* (SV78869). **B.** The invasion ratios (output bacteria/ input bacteria) of epithelial HeLa cells by four *Salmonella* strains are represented: the wild type (wt), a strain with a deletion of SPI-1 (*ΔSPI-1*), a strain carrying the P_L*tetO*_*-stdEF* construct, and a strain carrying the P_L*tetO*_*-*Δ*stdEF* construct. **C.** StdE binding to the *hilD* coding region. StdE-C and StdF-C indicate binding peaks found for the mock IP-sample.

Expression of *S*. *enterica* pathogenicity island (SPI-1) in the presence and in the absence of StdE and StdF was monitored using a *gfp* fusion constructed downstream of the SPI-1 gene *sipB*. A known trait of SPI-1 expression is bistability [43–45]. Single cell analysis of *sipB*::*gfp* expression showed that the SPI-1^ON^ subpopulation was absent in the P_L*tetO*_-*stdEF* strain (Fig 5, panel A). A concomitant observation was that invasion of epithelial cells was severely impaired in the P_L*tetO*_-*stdEF* strain (Fig 5, panel B).

ChIP-seq analysis revealed that StdE (but not StdF) binds SPI-1, mainly within the coding region of *hilD* (Fig 5, panel C). Minor binding peaks within SPI-1 were however found in the *sipB* and *sipC* genes (S2 Table). Outside SPI-1, no binding was detected in genes known to encode SPI-1 regulators (e. g., *hilE* and *rtsA*).

Downregulation of SPI-1 expression is in agreement with the occurrence of StdE-mediated postranscriptional repression of *hilD* [30], and binding of StdE to the *hilD* coding sequence suggests that StdE-mediated regulation may be direct. If such is the case, a tentative speculation is that StdE binding at the *hilD* coding region might interfere with the DNA-RNA hybrid formed during transcription, reducing the transcription rate. Binding of transcription factors to DNA-RNA hybrids has been previously described [46]. Another speculation is that binding of StdE might regulate the expression of either an antisense RNA or an RNA derived from the 3' untranslated region of *hilD* [47], resulting in a decrease of *hilD* mRNA.

#### Biofilm formation

Another gene downregulated by StdE/StdF, *ygiD*, has been shown to be involved in biofilm formation in both *Salmonella enterica* and *E*. *coli* [48,49]. On these grounds, we compared biofilm formation by strains P_L*tetO*_-*stdEF* (SV8141) and P_L*tetO*_-Δ*stdEF* (SV8142) using a standard method [50]. Biofilm formation was indeed reduced in strain P_L*tetO*_-*stdEF* as predicted by transcriptomic analysis (S3 Fig). However, StdE-F binding to the *ygiD* gene was not detected by ChIP-Seq (S2 Table), suggesting that regulation might be indirect. Further analysis was not pursued for this reason.

#### Conjugation

**Fig 6:**
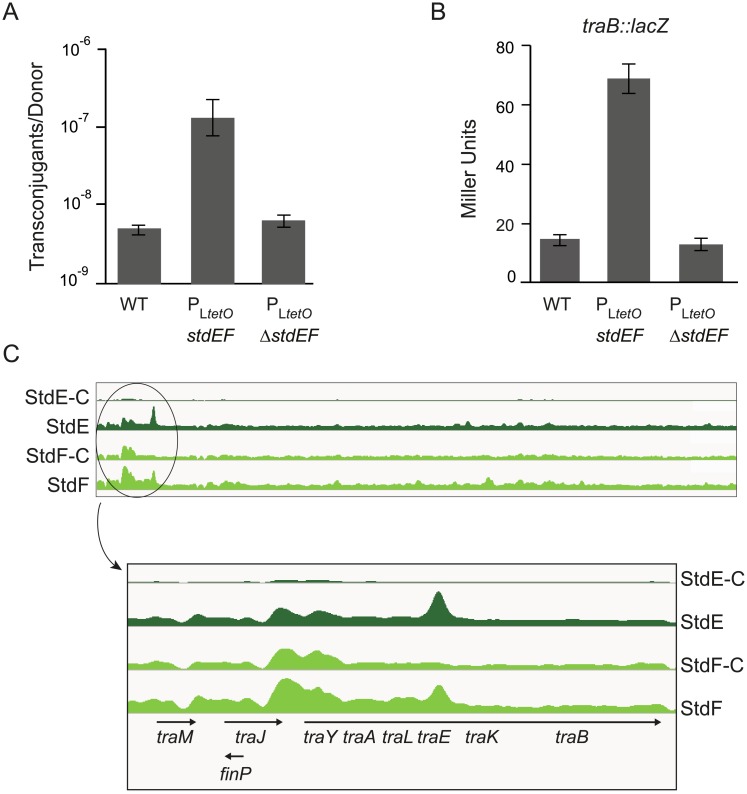
Regulation of conjugal transfer by StdE. **A.** Conjugal transfer of the virulence plasmid. Donors were the wild type strain, P_L*tetO*_-*stdEF spvA* (SV7554) and P_L*tetO*_-Δ*stdEF spvA* (SV7555). The recipient was *trg*::*mudQ* pSLT^-^ (SV4938) in all matings. Values are averages and standard deviations from 3 independent experiments. **B.** β-galactosidase activity of a *traB*::*lacZ* fusion in strains *traB*::*lacZ* (SV7551), P_L*tetO*_-*stdEF traB*::*lacZ* (SV7550), and P_L*tetO*_-Δ*stdEF traB*::*lacZ* (SV7549). **C.** ChIP-seq data for the *tra* operon of the virulence plasmid. In the lower part of the figure, the promoter-proximal *tra* region is enlarged. StdE-C and StdF-C indicate binding peaks found for the mock IP-sample.

When strain P_L*tetO*_-*stdEF* (SV8141) was used as a donor in matings with an appropriate recipient (SV4938), a 10 fold increase in the frequency of conjugal transfer of the virulence plasmid was observed (Fig 6, panel A). This increase in plasmid transfer was consistent with transcriptomic analysis (24 fold increase) and with upregulation of a *traB*::*lacZ* transcriptional fusion upon constitutive expression of StdEF (6 fold increase) (S1 Table and Fig 6, panel B). These observations are also consistent with ChIP-seq data showing StdE and StdF binding at the *traJ-traY-traA* region in the virulence plasmid (Fig 6, panel C). Binding is detected upstream of *traY*, within the region that contains the main *tra* promoter [51]. Binding within coding sequences also occurs (e. g., in *traE*, a region that does not contain any known promoter), a phenomenon previously described for other transcriptional regulators [38–40].

### Analysis of lineage-specific traits in sorted Std^OFF^ and Std^ON^ subpopulations

Considering that the *std* operon is expressed in a minor subpopulation of cells (Fig 1), the phenotypic traits detected in the StdEF^+^ strain can be expected to occur in a small fraction of cells only. To monitor the occurrence of lineage-specific traits, Std^ON^ and Std^OFF^ cell lineages were separated by two independent single cell techniques: fluorescence activated cell sorting (FACS) and magnetic activated cell sorting (MACS). A difference between these procedures is that FACS yields live cells while MACS yields fixed cells. The small size of the Std^ON^ lineage made these experiments challenging as sorting is unable to yield pure Std^ON^ and Std^OFF^ cell lineages. However, the size of the Std^ON^ lineage increased >230 fold (from 0.3% to 70%), thus permitting comparison with the Std^OFF^ lineage.

**Fig 7:**
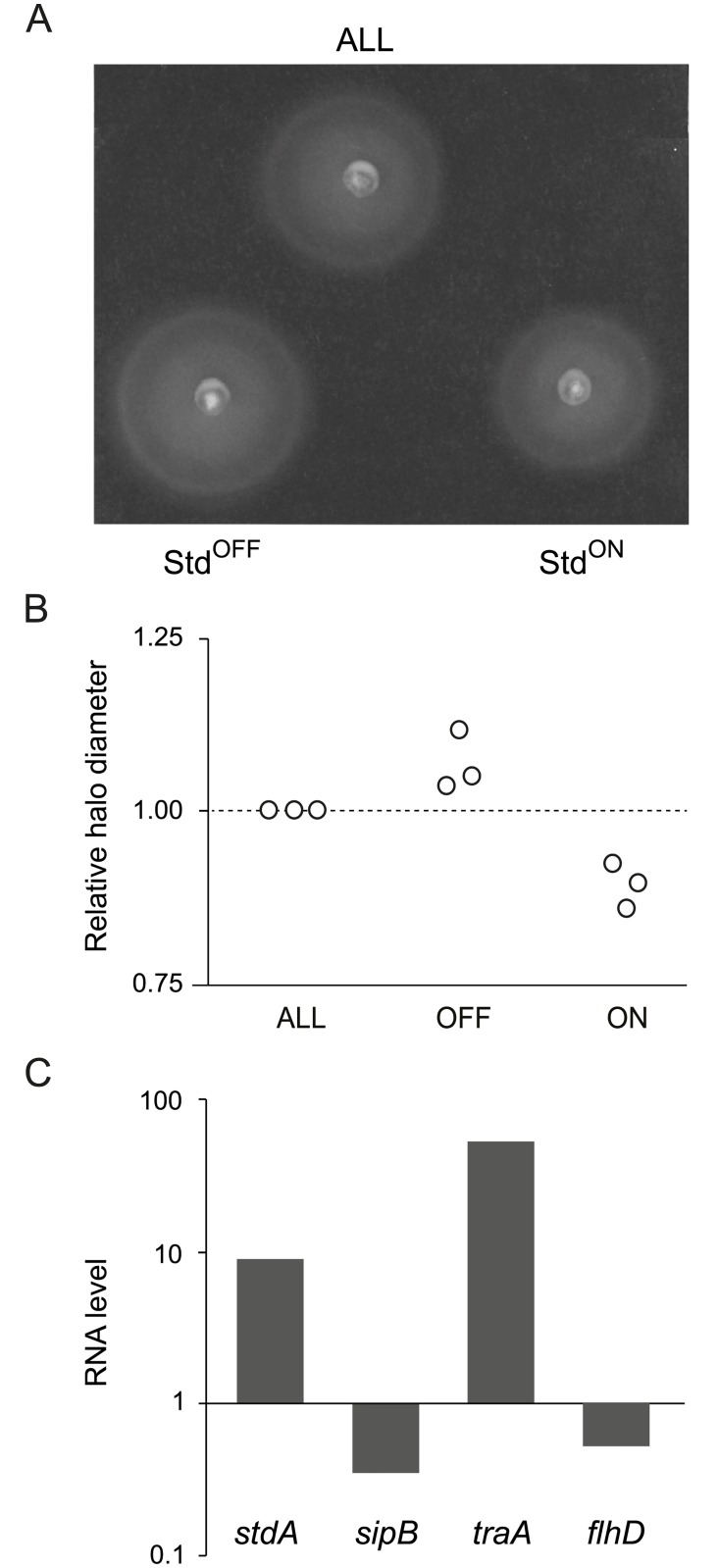
Analysis of lineage-specific traits upon cell sorting. **A.** Motility of sorted cells (Std^OFF^ and Std^ON^ subpopulations) and of the whole bacterial population (ALL). The experiments were performed in triplicate, and a representative experiment is shown. **B.** Relative diameter of motility halos from 3 independent experiments. Data are normalized to the halo diameter obtained in the whole bacterial population (ALL) which was set to "1" in all cases. **C.** RNA levels detected by RT-PCR upon magnetic-activated cell sorting (MACS) of Std^OFF^ and Std^ON^ cell lineages. For each locus, data are normalized to the RNA level obtained in the Std^OFF^ population (which was set to "1" in all cases). RNA levels were determined for 3 replicates, and a representative experiment is shown.

Motility assays on soft agar plates provided evidence that Std^ON^ cells show reduced motility as predicted by transcriptomic analysis (Fig 7A and 7B). In turn, the occurrence of lineage-specific transcriptional patterns was confirmed by RT-PCR analysis of *sipB*, *traA*, *and flhD* gene expression upon separation of Std^OFF^ and Std^ON^ cells by MACS (Fig 7C). The *stdA* gene was included as a control to validate cell separation (Fig 7C). The differences in the relative levels of *sipB*, *flhD* and *traA* mRNAs detected between sorted Std^OFF^ and Std^ON^ cells were consistent with the observations made upon constitutive expression of StdE and StdF (Fig 2).

## Discussion

The addition of the *std* operon of *S*. *enterica* to the list of bacterial loci that show bistable expression is far from exceptional: subpopulation formation is common among loci that encode envelope structures such as the flagellum [52], the O-antigen of the lipopolysaccharide [14,52] and fimbrial and non-fimbrial adhesins [53–55]. However, an unsuspected ability of the *std* operon is control of host genes. Constitutive expression of the StdE and StdF proteins, which are encoded by downstream genes in the *std* operon, brings in major changes in the transcriptome (Table 1). At most loci, StdE and StdF appear to be repressors of transcription (143 loci, Table 1). However, positive control by StdEF is also detected (27 loci, Table 1). Note that Table 1 includes only loci with differences in RNA content of 4-fold or higher; with a threshold of 2-fold differences, the number of downregulated loci would increase to 187, and the list of upregulated loci to 116 (GEO, accession GSE45488).

StdE and StdF are cytoplasmic proteins with DNA binding capacity (Fig 3), and a number of StdE and StdF binding sites are located at or near promoters of genes under StdE and/or StdF control (Figs 4, 5 and 6). The conclusion that StdE and StdF may exert direct transcriptional control is supported by the relatedness of StdE with the transcriptional activators GrlA from *E*. *coli* and CaiF from *Enterobacter cloacae* [30]. In turn, StdF is related to the transcriptional regulator SprB of *S*. *enterica* [30]. Phenotypic analysis confirmed that distinct transcriptomic profiles of StdEF^+^ and StdEF^−^strains resulted in phenotypic differences: the StdEF^+^ strain showed reduced motility (Fig 4), reduced invasion of epithelial cells (Fig 5), increased conjugal transfer (Fig 6), and reduced biofilm formation (S3 Fig).

Reduced motility of the fimbriated lineage, a feature reminiscent of the StdEF^+^ strain, was confirmed upon cell sorting (Fig 7A and 7B). The smaller difference in motility between Std^OFF^ and Std^ON^ cells may be explained by formation of Std^OFF^ cells during growth on motility agar. Note that growth can be expected to partially blur differences, and a long incubation time is required due to the small number of cells recovered by sorting. Hence, the real differences between Std^ON^ and Std^OFF^ lineages are probably larger than shown under the conditions of our experiments.

The occurrence of a lineage-specific transcriptional pattern in Std^ON^ cells was also confirmed by RT-PCR analysis upon separation of fimbriated and non-fimbriated cells by magnetic activated cell sorting (MACS) (Fig 7). Hence, it seems reasonable to conclude that the Std^ON^ lineage may display phenotypes similar or identical to those detected upon constitutive expression of StdE and StdF. Note than phenotypes other than motility were not tested in sorted Std^ON^ cells because the experiments required previous growth, which would yield a mixture of Std^OFF^ and Std^ON^ cells with outmost predominance of the Std^OFF^ lineage.

Adhesion to the cecal epithelium by Std fimbriae may adapt the Std^ON^ lineage to colonize the large intestine [28]. The size of the Std^ON^ lineage in the intestine remains unknown at this stage. However, a small Std^ON^ subpopulation may be sufficient for chronic infection, and production of Std^OFF^ cells can permit *Salmonella* shedding into the environment as in other types of chronic infection [56]. Formation of Std^ON^ and Std^OFF^ lineages may thus be interpreted as a division of labour that adapts the *S*. *enterica* Std^ON^ subpopulation to acute infection and the Std^OFF^ subpopulation to chronic infection. An alternative interpretation is that lineage formation may increase the chances of host colonization by undertaking two distinct strategies [7]. Whatever the case, the occurrence of additional lineage-specific traits may contribute to adaptation, and the adaptive value of some such traits can be envisaged. For instance, downregulation of flagellar synthesis in Std^ON^ cells may contribute to immune evasion and reduce the energetic burden of building a machinery that may be superfluous in cells attached to a surface. A similar argument may explain the adaptive value of downregulation of pathogenicity island 1 (SPI-1) in the Std^ON^ lineage as *Salmonella* does not invade epithelial cells in the large intestine, and the SPI-1 type 3 secretion apparatus is a costly structure [45]. Activation of the *tra* operon of the virulence plasmid is a more enigmatic trait. However, high rates of pSLT conjugal transfer have been previously detected in the mammalian intestine [57]. The potential adaptive value of inhibition of biofilm formation in the Std^ON^ lineage cannot be understood at this stage. Speculation on the existence of additional lineage-specific phenotypic differences would be likewise premature. However, the high number of genes under StdE and StdF control revealed by transcriptome analysis suggests that such differences may exist.

Pleiotropy may be an unusual capacity of a fimbrial locus. However, additional examples of pleiotropic switching have been described in the bacterial world. Phase variation in beta-hemolytic properties in the dental pathogen *Streptococcus gordonii* is accompanied by changes in adhesive properties and surface antigens [58]. In *Pseudomonas aeruginosa*, small colony variants show increased biofilm formation and impaired motility and chemotaxis [59]. In a *Salmonella* strain that causes asymptomatic infection in pigs, phase variation of type I fimbriae is accompanied by changes in adhesion, uptake by phagocytes, and survival within phagocytes [60]. Colony variants of *Haemophilus influenzae* also differ in multiple surface proteins [61]. While these studies were mostly descriptive, a molecular mechanism that may exert pleiotropic control of gene expression has been described in the phasevarions of *Neisseria*, *Campylobacter*, *Haemophilus*, and other bacterial pathogens [62,63]. In a phasevarion, bistable expression of a DNA methyltransferase gene gives rise to bacterial lineages that differ in the presence or the absence of methylation at multiple genome locations. As a consequence, the lineages may differ in the expression of loci sensitive to the methylation state of their promoters. If transcriptional control by DNA methylation occurs in multiple genes, a tentative analogy with the StdE/StdF pleiotropic switch can be drawn.

In *Salmonella*, sequential acquisition of pathogenicity islands and other genetic determinants has enabled the pathogen to colonize animals [48,64], and the accommodation of such entities in the host regulatory network has been made possible by complex transcriptional and postranscriptional controls [65]. In the case of *std*, accommodation in the host regulatory network is known to be exerted by Dam methylation and HdfR [31], and additional controls may exist. However, reciprocal control of host loci by the *std* operon introduces an interesting twist into the contribution of horizontal transfer to *Salmonella* evolution. Regulation of host genes has been described in prophages [66,67] and in plasmids [68–70]. Occurrence of an analogous capacity in a small genetic entity devoid of autonomous lifestyle is remarkable, especially if one considers the high number of genes under StdEF control.

## Methods

### Bacterial strains, bacteriophages and strain construction

*Salmonella enterica* strains listed in S3 Table belong to serovar Typhimurium and derive from the mouse-virulent strain SL1344 [71]. For simplicity, *Salmonella enterica* serovar Typhimurium is often abbreviated as *S*. *enterica*. *E*. *coli* BL21 [F^−^*dcm ompT hsdS* (rB^−^mB^–^) *gal* [malB^+^]K12(λS)] (Stratagene, La Jolla, CA) was used for protein purification. Targeted gene disruption was achieved using plasmids pKD3, pKD4 or pKD13 as templates to generate PCR products for homologous recombination [72]. Antibiotic resistance cassettes introduced during strain construction were excised by recombination with plasmid pCP20 [72]. Addition of a 3xFLAG epitope tag to the *stdA* coding sequence was carried out using plasmid pSUB11 as template [73]. Primers used in strain construction are shown in S4 Table. Transductional crosses using phage P22 HT 105/1 *int201* [74] were used for strain construction operations involving chromosomal markers. The transduction protocol has been previously described [75]. To obtain phage-free isolates, transductants were purified by streaking on green plates. Phage sensitivity was tested by cross-streaking with the clear-plaque mutant P22 H5. Construction of strains SV9324 (Δ*dam*231 *stdEF*::3xFLAG) and SV9325 (Δ*dam*231 *stdF*::3xFLAG) was performed by transductional crosses from SV6749 and SV6502 [30] to JH3294 (Δ*dam*231) [76]. Construction of strain SV9287 (P_L*tetO*_*-stdEF*::3xFLAG) was performed by transduction from SV6510 [30].

For construction of the transcriptional *gfp* fusion of strain SV9597 (*stdA*::*gfp*), a fragment containing the promoterless green fluorescent protein (*gfp*) gene and the chloramphenicol resistance cassette was PCR-amplified from pZEP07 (Hautefort et al., 2003) using oligonucleotides stdASTOPGFP P1 and stdAGFP P2 (S4 Table). The fragment was integrated into the chromosome of *S*. *enterica* [72], and integration was verified using oligonucleotides *stdA E1* and *stdA E2* (S4 Table). The Cm^R^ resistance cassette was changed to Km^R^ using oligos Cm-P1-R and FliC-GFP-Km-P4, and later excised with plasmid pCP20 [72].

Construction of SV8141 (P_L*tetO*_*-stdEF*) and SV8142 (P_L*tetO*_*-*Δ*stdEF*) was performed by transducing the wild type with lysates from SV6503 (P_L*tetO*_*-stdEF*) and SV6634 (P_L*tetO*_*-*Δ*stdEF)* [30]. In both constructions, the P_L*tetO*_ promoter is inserted upstream of *stdE* on the *Salmonella* chromosome. In both strains, the upstream *std* genes and the promoter of *stdA* are deleted. A Cm^R^ cassette in reverse orientation linked to the P_L*tetO*_ promoter provided a selectable marker. For construction of SV7553 and SV7552, the Cm^R^ resistance gene was replaced with a Km^R^ cassette amplified from pKD13 [72] with oligos Km P_L*tetO*_ P1 and Km P_L*tetO*_ P2 (S4 Table). The resulting PCR product was transformed into strains SV8141 (P_L*tetO*_*-stdEF*) and SV8142 (P_L*tetO*_*-*Δ*stdEF)* containing pKD46 [72]. Km^R^ colonies were selected on LB + kanamycin. Finally, the Km^R^ cassette introduced during construction was excised by recombination with plasmid pCP20 [72].

### Media and growth conditions

Bertani’s lysogeny broth (LB) was used as standard rich medium. Solid media contained agar at 1.5% final concentration. Cultures were grown at 37°C. Aeration of liquid cultures was obtained by shaking at 200 rpm in an Infors Multitron shaker. Antibiotics were used at the final concentrations described elsewhere [77].

### RNA isolation, microarray procedures, and data analysis

To prepare cells for RNA extraction, 3 ml of fresh LB was inoculated with a 1:100 dilution from an overnight bacterial culture, and incubated with shaking. A 2 ml aliquot from a stationary culture (OD_600_≅2) was centrifuged at 13,000 rpm, 4°C, during 5 min. The pellet was then resuspended in 100 μl of lysozyme (3 mg/ml in water; Sigma Chemical Co.), and cell lysis was facilitated by a freeze-thaw cycle. After lysis, RNA was extracted using 1 ml of TRIsure reagent following manufacture’s instructions (Bioline, Taunton, Massachusetts, USA). Total RNA was resuspended in 150 μl of RNase-free water, and subsequently cleaned by extraction with acidic phenol, followed by a second extraction with chloroform:isoamilic alcohol (24:1). After extraction, RNA was precipitated with ethanol and 3 M sodium acetate, and the dried pellet was resuspended in RNase-free water. The quantity and quality of the RNA was determined using a ND-1000 spectrophotometer (NanoDrop Technologies).

Transcriptomic analyses were performed using the *Salmonella enterica* serovar Typhimurium SL1344 4X72K array [35]. Hybridation and microarray scanning were performed at the Functional Genomics Core of the Institute for Research in Biomedicine, Baldiri Reixac, Barcelona, Spain (http://www.dnaarrays.org/). Normalization of the expression signals was done with RMA (Irizarry et al., 2003) using Partek Genomics suite 6.5 (6.11.0207). Raw transcriptomic data were deposited at the Gene Expression Omnibus, G.E.O, database (http://www.ncbi.nlm.nih.gov/geo/) under accession number GSE45488. Differential gene expression was assessed using the Limma’s R package [78]. Background correction and normalization of gene expression were done using RMA algorithm [79]. A gene was considered significant for a Benjamini and Hochberg-corrected (BH) p-value of <0.05 in a moderated t-statistic and a log2 fold change > 2. Gene identities were annotated according to the *S*. *enterica* ser. Typhimurium strain SL1344 genome sequence (ftp://ftp.sanger.ac.uk/pub/pathogens/Salmonella/STmSL1344.dbs). RNA isolation from ~10^6^ *S*. *enterica* cells sorted by MACS was performed using Direct-zol RNA MiniPrep (Zymo Research, Irvine, California, USA), following manufacturer’s instructions.

### Quantitative reverse transcriptase PCR (qRT-PCR)

For quantitative RT-PCR, *Salmonella* RNA was extracted from stationary phase cultures (OD_600_≅2) and from sorted cells as described above, and the concentration was determined using a ND-1000 spectrophotometer (NanoDrop Technologies). An aliquot of 1 μg of RNA was used for cDNA synthesis using QuantiTec Reverse Transcription Kit (Quiagen) following manufacturer’s instructions. Quantitative RT-PCR reactions were performed in a Light Cycler 480 II apparatus (Roche). Reactions were carried out in a total volume of 10 μl on a 480-well optical reaction plate (Roche), using Takara SYBR Premix Ex Taq reagent. Each reaction contained 4μl cDNA (1/10 dilution), 5 μl of 2X SYBR mix, 0.2 μl DYE II, and two gene-specific primers at a final concentration of 0.2 mM each. Real-time cycling conditions were as follows: (i) 95°C for 10 min and (ii) 40 cycles at 95°C for 15 s, 60°C for 1 min. A non-RT control (without reverse transcriptase) was included for each primer set. Triplicates were run for each reaction, and the Ct value is averaged from them. Absence of primer dimers was corroborated by running a dissociation curve at the end of each experiment to determine the melting temperature of the amplicon. Melting curve analysis verified that each reaction contained a single PCR product. Gene-specific primers were designed with ProbeFinder software (http://www.universalprobelibrary.com) from Roche Applied Science, and are listed in S4 Table.

For quantification, the efficiency of each primer pair was determined to be between 90%-110%, following the instructions for efficiency determination described in the “Guide to Performing Relative Quantification of Gene Expression Using Real-Time Quantitative PCR” (Applied Biosystems). Relative RNA levels were determined using the ΔΔCt method as described in the same guide. Each ΔΔCt determination was performed at least in three different RNA samples.

### Purification of the StdE protein and antibody generation

A DNA fragment containing *stdE* was amplified using oligonucleotides NdeIstdE-FOR and EcoRIstdE-REV, and cloned into *Nde*I- and *Eco*RI-digested pET28a (Novagen). The recombinant plasmid (pIZ1991) was verified by restriction analysis and DNA sequencing. For 6×His-StdE purification, plasmid pIZ1991 was transformed into *E*. *coli* BL-21. BL-21/pIZ1991 was grown in LB broth containing kanamycin, and expression of 6×His-StdE was induced with 1 mM isopropyl β-D-thiogalactopyranoside (IPTG). After 3 h induction, cells were centrifuged and resuspended in 10 ml of lysis buffer (20 mM Tris, 300 mM NaCl, 10 mM imidazole) per g of pelleted cells, and were lysed by sonication with 4 cycles of 30 seconds. The suspension was centrifuged at 10,000 rpm for 20 min at 4°C and the supernatant containing the soluble fraction of 6×His-StdE was transferred to a HisTrap HP nickel affinity chromatography column (GE Healthcare, Wauwatosa, WI, USA). The column was washed 3 times with 4 ml of washing buffer (20 mM NaH_2_PO_4_∙H_2_O, 0,5 mM NaCl, 30 mM imidazole). Protein elution was performed with 3 ml of elution buffer (20 mM NaH_2_PO_4_∙H_2_O, 0.5 mM NaCl, 300 mM imidazole). Elution fractions enriched in 6×His-StdE were selected. Imidazole was removed by dialyzing in cellulose membranes with PBS 1X. Purified 6×His-StdE protein was sent to Biomedal S.L (Sevilla, Spain) for polyclonal antisera production in rabbits. The working dilution was prepared based on manufacturer’s recommendations.

### Chromatin immunoprecipitation followed by sequencing (ChIP-seq) and data analysis

Strain P_L*tetO*_*-stdEF*::3xFLAG was used to perform ChIP-seq experiments. Twenty ml of fresh LB was inoculated with a 1:100 dilution from an overnight bacterial culture, and incubated with shaking at 200 rpm at 37°C. Cells collected at OD_600_≅2 were cross-linked with 1% formaldehyde at 37°C for 25 min, followed by quenching of the unused formaldehyde with 450 mM glycine for an additional incubation of 5 min. Cross-linked cells were harvested and washed with 10 ml of TBS pH7.6 (2.42 g/l Trizma base, 8 g/l NaCl). The washed cells were resuspended in 1 ml of lysis buffer (10 mM Tris-HCl pH 8, 20% sucrose, 50 mM NaCl, 10 mM EDTA) and after an additional centrifugation step, the cells were resuspended in 0.5 ml of lysis buffer with lysozyme (20 mg/ml; Sigma Chemical Co.). The cells were incubated for 30 min at 37°C and then treated with 4 ml of IP buffer (50 mM HEPES-KOH pH7.5, 150 mM NaCl, 1 mM EDTA, 1% Triton X-100, 0.1% Na deoxycholate, 0.1% SDS and 1mg/ml PMSF). The lysate was then sonicated using a Bioruptor (Diagenode) with 5 cycles of 7 minutes at high setting. Cell debris was removed by centrifugation for 20 min at 4°C and the supernatant was used as a cell extract for immunoprecipitation. The range of fragment sizes resulting from sonication was 100–500 bp, and the average fragment size was 300 bp (S2 Fig).

To immunoprecipitate StdE-DNA and StdF-DNA complexes, 800 μL of chromatin, 20 μL of Ultralink Immobilized protein A/G beads (Pierce) and 2 μL of the corresponding antibody were used. A control sample (mock-IP) with no antibody was included. Four samples were used for each antibody and four samples for the control. Incubation for 90 min was performed at room temperature on a rotating wheel. Beads were transferred to a Spin-X column tube (Costar) and centrifuged at 3,000 rpm for 1 min. Beads were gently re-suspended in 500 μL of IP buffer and incubated on the wheel for additional 3 min. This step was done twice. Beads were washed with 500 μL of IP salt buffer, IP wash buffer and TE pH 8.0 by resuspending and centrifuging the sample. The column was transferred to a fresh tube and the beads were resuspended in 100 μL of elution buffer (50 mM Tris-HCl at pH 7.5, 10 mM EDTA, and 1% SDS) and incubated at 65°C for 20min. After centrifuging at 3,000 rpm for 1 min, the flow-through was treated with 10 μL of 40 mg/ml Pronase (Roche) made up in TBS. The samples were heated at 42°C for 2h and 65°C for 6 h. The reactions were then kept at 4°C overnight. The samples were cleaned using a PCR clean-up Kit (Promega) and resuspended in 50 μL of H_2_O.

Input and ChIP DNA samples were sent for sequencing at the Functional Genomics Core Facility of the Institute for Research in Biomedicine, Barcelona (Spain). Next generation sequencing was carried out using Illumina’s sequencing technology. Ultra DNA Library Prep Kit (Illumina) was used for library preparation. Libraries were sequenced on Illumina’s Genome Analyzer II system. 50 nucleotides single end reads were obtained following strictly manufacturer’s recommendations. Illumina sequencing data were pre-processed with the standard Illumina pipeline version 1.5. BAM files reported by the sequencing facility were converted to FASTQ format with the BAM2FASTQ tool (https://gsl.hudsonalpha.org/information/software/bam2fastq).The quality of the sequence reads was examined using FASTQC [80] that reported the presence of Illumina adapters. The adapters were trimmed with the FASTX_CLIPPER tool of the FASTX-Toolkit suite (http://hannonlab.cshl.edu/fastx_toolkit/). Reads shorter than 40 nt were discarded. NCBI GCA_000210855.2 genome assembly of *S*. *enterica* SL1344 was used as reference genome. Mapping was performed with Bowtie [81] allowing two-mismatches for only unique alignment. Peaks were called using CisGenome version 2.0 [82] using default parameters. The IGV browser [83] was used for data visualization. Genes closest to a ChIP peak were identified using the bedtools suite [84]. Peak boundaries sequences were extracted from the reference genome using the fastaFromBed utility from the BEDTools suite (E) and analyzed with DREME [85] for motif discovery.

### β-galactosidase assays

Levels of β-galactosidase activity were determined using the CHCl_3_-sodium dodecyl sulfate permeabilization procedure [86]. β-galactosidase activity data (Miller Units) are the averages and standard deviations from ≥3 independent experiments.

### Infection of epithelial cells

HeLa human epithelial cells (ATCC CCL2) were grown in DMEM containing 10% fetal calf serum and 1mM glutamine (Life Technologies). The day before infection, approximately 10^5^ HeLa cells were seeded, using 24-well plates (Costar, Corning, New York, NY) containing 1 ml of tissue culture medium without antibiotics per well, and grown at 37°C, 5% CO_2_ to obtain 80% confluency. One hour before infection, the culture medium was removed and replaced by 0.5 ml fresh tissue culture medium without antibiotics. Bacteria were grown overnight at 37°C in LB with shaking, diluted into fresh medium (1:50), and incubated at 37°C without shaking up to O.D._600_ 0.6–0.8 (overnight). Bacteria were added to reach a multiplicity of infection (MOI) of 50:1 bacteria/HeLa cell. HeLa cells were infected for 30 min, washed 3 times with PBS, incubated in fresh tissue culture medium containing 100 μg/ml gentamicin for 1.5 h, and washed 3 times with PBS. Numbers of viable intracellular bacteria were obtained by lysing infected cells with 1% Triton X-100 (prepared in PBS) and subsequent plating. Invasion rates were determined as the ratio of viable intracellular bacteria vs. viable bacteria added to infect the HeLa cells.

### Motility assays

Motility assays were carried out on motility agar plates containing 10 g/l tryptone (Difco), 5 g/l NaCl, and 0.25% bacto-agar [87]. A sterile stick was soaked in saturated bacterial cultures grown in LB, and used to inoculate motility agar plates. Bacterial motility halos were compared after growth at 37°C for 6 h. For motility assays of sorted cultures, the incubation time was 12-18h. A simultaneous viability test was performed by plating on LB, to warrant that the number of cells was the same for each subpopulation.

### Biofilm formation

Biofilm formation was tested in LB medium [50]. Cultures were grown at 22°C for 7–10 days. For better visualization, the biofilm was stained with a 0.1% solution of crystal violet.

### Matings

Cultures of the donor and the recipient were grown overnight in LB broth. Cells were harvested by centrifugation and washed with LB. Aliquots of both strains, 1ml each, were sucked onto a membrane filter with a 0.45 μm pore size with a donor/recipient ratio of 1:1. The filters were then placed on LB plates and incubated during 4 h at 37°C in a GasPak microaerophilic jar [88]. Conjugation frequencies were calculated per donor cell as previously described [57,88].

### Immunofluorescence microscopy

Cells from 1.5 ml of an exponential culture (OD_600_≅0.5) were collected by centrifugation, washed, resuspended in 1 ml TE buffer and fixed by adding the same volume of cold 70% ethanol. Ethanol-fixed cells (100 μl) were stained with polyclonal rabbit anti-StdA serum 1:250 [89]. After extensive washing with PBS + gelatin 0.02%, goat anti-rabbit antibody conjugated to FITC (fluorescein isothiocynate, 1:500) was used. Immunostained cells were placed in 10 μl mounting medium (40% glycerol in 0.02 M phosphate buffered saline, pH 7.5). 20 μl of ethanol-fixed cells were spread onto a poly-L-lysine-coated slide, and dried at room temperature. Slides of stained samples were stored at room temperature in the dark. Images were obtained by using an Olympus IX-70 Delta Vision fluorescence microscope (Olympus, Tokyo, Japan) equipped with a 100X UPLS Apo objective. Pictures were taken using a CoolSNAP HQ/ICX285 camera (Roper Technologies, Sarasota, FL) and analysed using ImageJ software (Wayne Rasband, Research Services Branch, National Institute of Mental Health, MD).

### Flow cytometry analysis

Bacterial cultures were grown at 37°C in LB until stationary phase (OD_600_≅2). Cells were then diluted in PBS to a final concentration of ~10^7^ cells/ml. Data acquisition and analysis were performed using a Cytomics FC500-MPL cytometer (Beckman Coulter, Brea, CA). Data were collected for 100,000 events per sample and were analysed with CXP and FlowJo 8.7 softwares. Data are shown by dot plots (forward scatter [cell size] *vs* fluorescence intensity).

### Fluorescence activated cell sorting (FACS) of live cells

Stationary cultures were washed and resuspended in PBS to a final concentration of 5 × 10^6^ cells/ml. Cells were sorted using a MoFlo Astrios EQ cytometer (Beckman Coulter, Brea, CA). Immediately before sorting, 5 × 10^6^ cells were analyzed for GFP expression. Based on this analysis, gates were drawn to separate the ~0.3% of cells expressing GFP (Std^ON^ state) from the ~99.7% of non expressing GFP cells (Std^OFF^ state). The whole population (ALL) was also analyzed after FACS.

### Magnetic activated cell sorting (MACS)

Five hundred ml from a stationary culture (OD_600_≅2) of strain SV9600 (*stdA*::3XFLAG) grown at 37°C with shaking were collected by centrifugation. The pellet was washed with 10 ml of TE buffer and fixed by adding the same volume of cold 70% ethanol. Ethanol-fixed cells were washed with PBS containing 0.05% of Tween (PBS-T). This step was repeated three times. The pellet was resuspended in 5 ml of lysozyme solution (2 mg/ml lysozyme, 25 mM Tris-HCl pH 8.0, 50 mM glucose and 10 mM EDTA) and incubated at room temperature for 10 min. Cells were washed 3 times with PBS-T and resuspended and incubated for 30 min in 10 ml of 2% BSA made up in PBS-T. After collecting cells by centrifugation, anti-flag-PE antibody (Miltenyi Biotec S.L.) was added. After 1 h of incubation at room temperature and extensive PBS-T washing, anti-PE microbeads (Miltenyi Biotec S.L.) were added and incubated overnight at 4°C followed by 3 PBS-T washing. Separation of labelled and unlabelled cells was performed using an autoMACS Pro Separator (Miltenyi Biotec S.L.).

## Supporting information

S1 Table Microarray data. (PDF)

S2 Table ChIP_seq data. (PDF)

S3 Table Strain list. (PDF)

S4 Table List of oligonucleotides. (PDF)

S1 Fig Microarray strains. (PDF)

S2 Fig Quality control ChIP-seq. (PDF)

S3 Fig Biofilm. (PDF)
