# Supplementary material for: Formation of phenotypic lineages in Salmonella enterica by a pleiotropic fimbrial switch
Source: PLoS Genet. 2018 Sep 25;14(9):e1007677. doi: 10.1371/journal.pgen.1007677 (PMC6173445; doi:10.1371/journal.pgen.1007677)
Supplement: S1 Table — (PDF) [file pgen.1007677.s001.pdf]

Table S1. Loci regulated by StdEF (log_2 fold change >2)

Locus number	Gene (primary)	Gene (synonym)	BH	Log_2 Fold Change	Description
SL0982			4.64876E-06	+6.23	Predicted bacteriophage protein
SLP1_0032	*traA*		3.32862E-05	+4.63	Conjugative transfer: fimbrial subunit
SLP1_0033	*traY*		0.000167528	+3.97	Conjugative transfer oriT nicking protein
SL3102	*ordL*		3.50667E-05	+3.85	Hypothetical oxidoreductase
SL3103			0.000310224	+3.73	Possible aldehyde dehydrogenase
SL0192	*fhuA*		0.000721465	+3.54	Ferrichrome-iron receptor
SLP1_0031	*traL*		0.00023774	+3.44	Conjugative transfer assembly protein
SL1277	*nlpC*		6.09217E-05	+3.42	Hypothetical lipoprotein
SLP1_0030	*traE*		0.000211619	+3.41	Conjugative transfer assembly protein
SL3858	*yifA*	*hdfR*	0.000423401	+3.05	HTH-type transcriptional regulator HdfR (H-NS-dependent flhDC regulator)
SL3101			9.81088E-05	+3.04	Uncharacterized protein
SL3143			0.000229778	+2.84	Hypothetical membrane transport protein
SL3144			0.001766587	+2.81	Possible membrane transport protein
SL3097			0.000760843	+2.77	Arylsulfatase regulator
SLP1_0027	*traP*		0.000296119	+2.68	Conjugative transfer protein
SL4480			0.001193366	+2.58	Uncharacterized protein
SL3100			0.002671684	+2.52	Possible amino acid transport protein
SL3145	*ygiK*		0.000478896	+2.35	Decarboxylate transporter
SL3653			0.001149024	+2.29	Uncharacterized protein
SLP2_0079	*traE*		0.006734973	+2.27	Conjugal transfer protein
SL3096			0.002157547	+2.23	Arylsulfatase
SL1062	*putA*		0.001442561	+2.11	Bifunctional protein PutA
SL3099			0.012767655	+2.11	Uncharacterized protein

SL0502		0.002044914	+2.10	Outer membrane protein
SL2841	*sitA*	0.013238408	+2.06	Iron transport protein, periplasmic-binding protein
SL0576	*entF*	0.014983519	+2.04	Enterobactin synthetase component F
SL4479	*bglJ*	0.011527588	+2.00	Conserved hypothetical regulatory protein
SL1527	*ydcX*	0.005577498	-2.05	Hypothetical inner membrane protein
SL1896		0.000792541	-2.09	Hypothetical exported protein
SL3213	*tdcE*	0.005880343	-2.13	Probable formate acetyltransferase
SL1867		0.000617276	-2.18	Hypothetical lipoprotein
SL2866	*spaS*	0.000450771	-2.26	Type III secretion system secretory apparatus
SL3163	*ygiD*	0.00282481	-2.26	Uncharacterized protein
SL0776	*slrP*	0.000465731	-2.36	Type III secretion system effector protein
SL2511	*asrB*	0.002399158	-2.45	Anaerobic sulfite reductase subunit B
SL0758	*modA*	0.000296119	-2.45	Molybdate-binding periplasmic protein
SL0759	*modB*	0.000304995	-2.47	Molybdenum transport system permease protein ModB
SL1849	*flhB*	0.000515773	-2.51	Flagellar biosynthetic protein FlhB
SL1177		0.009862028	-2.52	Predicted bacteriophage protein
SL1027	*pipB*	0.000634226	-2.56	Type III secretion system effector protein, homologous to pipB2
SL3125	*yghW*	0.000546934	-2.58	Uncharacterized protein
SL2510	*asrA*	0.00054414	-2.63	Anaerobic sulfite reductase subunit A
SL1879	*sdiA*	0.000404329	-2.65	Cell-division regulatory protein
SL2849	*orgB*	0.001266513	-2.70	Oxygen-regulated invasion protein OrgB
SL1028		0.000296119	-2.71	Conserved hypothetical inner membrane protein
SL1210	*yeaQ*	0.005723272	-2.72	Hypothetical inner membrane protein
SL1236		0.000245458	-2.78	Hypothetical MutT-family protein
SL0760	*modC*	0.000176572	-2.78	Molybdenum import ATP-binding protein ModC
SL4247		0.000193791	-2.82	Hypothetical membrane protein
SL2521	*cadA*	0.000156471	-2.87	Lysine decarboxylase

SL3569			0.000272039	-2.90	Hypothetical membrane protein
SL1235			0.000310224	-2.91	Hypothetical exported protein
SL2845	*avrA*		0.000130738	-2.95	Type III secretion system effector protein-regulator of Salmonella-induced inflammatory response
SL2858	*sptP*		0.000119998	-2.95	Type III secretion system effector protein
SL1904	*fliL*		0.003045139	-3.02	Flagellar protein FliL
SL3128			0.00041399	-3.03	Exported protein
SL2759	*iroC*		0.000949568	-3.03	Hypothetical ABC transporter protein
SL2520	*cadB*		0.000156471	-3.04	Probable cadaverine/lysine antiporter
SL1559	*steB*		0.000313925	-3.18	Type III secretion system effector protein
SL3129			0.000255607	-3.25	Uncharacterized protein
SL1784	*sopE2*		0.000211619	-3.28	Guanine nucleotide exchange factor
SL4248			0.000270946	-3.32	Predicted bacteriophage protein
SL1110	*flgA*		5.11706E-05	-3.58	Flagella basal body P-ring formation protein FlgA
SL3543	*yhhP*	*tusA*	0.000145167	-3.58	Sulfur carrier protein TusA (Sulfur mediator TusA) (Sulfur transfer protein TusA)
SL2859	*sicP*		5.89912E-05	-3.60	Chaperone protein sicP
SL2867	*spaR*		0.000296119	-3.62	Type III secretion system secretory apparatus
SL2758	*iroB*		6.34417E-05	-3.66	Hypothetical glycosyltransferase
SL2848	*orgC*		0.000199402	-3.75	Uncharacterized protein
SL4198	*siiF*		0.000515773	-3.78	Hypothetical type-1 secretion protein
SL2846	*sprB*		0.000465731	-3.81	AraC-family transcriptional regulator
SL1526	*srfC*		0.000123004	-3.88	Virulence protein
SL1860	*flhD*		0.000132585	-3.92	Flagellar transcriptional regulator FlhD
SL3130			9.36671E-05	-3.94	Uncharacterized protein
SL1525	*srfB*		2.84041E-05	-4.11	Virulence protein
SL1109	*flgM*		0.000171594	-4.14	Negative regulator of flagellin synthesis (Anti-sigma factor)
SL1859	*flhC*		0.000245458	-4.15	Flagellar transcriptional regulator FlhC

SL4249			2.35527E-05	-4.15	Uncharacterized protein
SL4250	*rtsB*		3.50667E-05	-4.15	Regulator of flhDC
SL1897	*fliE*		8.3145E-05	-4.16	Flagellar hook-basal body complex protein FliE
SL2868	*spaQ*		0.000843745	-4.18	Type III secretion system secretory apparatus
SL1848	*flhAa*	*flhA*	5.25264E-05	-4.20	Flagellar biosynthesis protein FlhA
SL1901	*fliI*		0.00019733	-4.25	Flagellum-specific ATP synthase
SL2869	*spaP*		0.001715648	-4.31	Type III secretion system secretory apparatus
SL2855	*hilD*		1.81005E-05	-4.32	Transcriptional regulator hilD
SL1083	*ymdA*		2.5867E-05	-4.33	Uncharacterized protein
SL4195	*siiC*		2.50404E-05	-4.35	Hypothetical type-I secretion protein
SL4193	*siiA*		0.000212322	-4.48	Type I secretion-related protein
SL1898	*fliF*		2.07036E-05	-4.51	Flagellar M-ring protein
SL2043	*sopA*		1.77215E-05	-4.52	Type III secretion system effector protein SopA-required for invasion and bacterial escape from the SCV
SL1524	*srfA*		2.06819E-05	-4.54	Virulence protein
SL2847	*sirC*	*hilC sprA*	9.9891E-05	-4.56	Transcriptional regulator sirC
SL1907	*fliO*		2.97973E-05	-4.57	Flagellar protein
SL1903	*fliK*		1.83702E-05	-4.68	Flagellar hook-length control protein
SL1847	*flhE*		7.68873E-05	-4.69	Flagellar protein FlhE
SL1900	*fliH*		8.03458E-05	-4.79	Flagellar assembly protein FliH
SL2876	*invE*		0.000115817	-4.85	Cell invasion protein
SL2854	*prgH*		4.87104E-05	-5.21	Type III secretion apparatus component
SL1117	*flgH*		1.74762E-05	-5.39	Flagellar L-ring protein (Basal body L-ring protein)
SL4194	*siiB*		2.09265E-05	-5.41	Hypothetical integral membrane protein
SL1108	*flgN*		1.2236E-05	-5.47	Flagella synthesis protein FlgN
SL4251	*rtsA*		6.44603E-06	-5.57	Regulator of SPI-1
SL1118	*flgI*		9.08515E-06	-5.58	Flagellar P-ring protein (Basal body P-ring protein)
SL1899	*fliG*		3.0918E-05	-5.62	Flagellar motor switch protein FliG

SL3126		3.28215E-05	-5.69	Methyl-accepting chemotaxis protein
SL1119	*flgJ*	2.27469E-05	-5.69	Flagellar protein FlgJ
SL1906	*fliN*	2.09588E-05	-5.73	Flagellar motor switch protein FliN
SL2879	*invH*	2.03828E-05	-5.80	Invasion lipoprotein invH
SL4196	*siiD*	1.81005E-05	-5.90	Hypothetical type-I secretion protein
SL1905	*fliM*	4.66107E-06	-5.92	Flagellar motor switch protein FliM
SL2924	*sopD*	5.53892E-06	-5.93	Type III secretion system effector protein-involved in bacterial invasion
SL4197	*siiE*	1.82855E-05	-5.96	Large repetitive protein
SL1263		1.85843E-05	-5.96	Hypothetical outer membrane protein
SL3112		4.77054E-06	-6.17	Uncharacterized protein
SL1884	*fliZ*	5.11706E-05	-6.21	FliZ protein
SL1726	*ycgR*	1.05997E-05	-6.25	Flagellar brake protein YcgR
SL2857	*iagB*	1.46423E-05	-6.26	Invasion protein iagB
SL2875	*invA*	7.89573E-06	-6.27	Secretory apparatus of type III secretion system
SL1888	*fliC*	7.88967E-06	-6.30	Flagellin
SL1853	*cheR*	9.10819E-06	-6.39	Chemotaxis protein methyltransferase (EC 2.1.1.80)
SL2865	*sicA*	6.44603E-06	-6.39	Type III secretion-associated chaperone
SL1850	*cheZ*	2.19221E-05	-6.42	Protein phosphatase CheZ (Chemotaxis protein CheZ)
SL2873	*invC*	2.03828E-05	-6.43	Secretory apparatus ATP synthase (Associated with virulence)
SL3576	*yhjH*	6.65E-06	-6.44	Uncharacterized protein
SL3190	*aer*	4.46595E-06	-6.45	Aerotaxis receptor protein
SL1891	*fliT*	4.02354E-06	-6.45	Flagellar protein FliT
SL1852	*cheB*	1.46423E-05	-6.46	Chemotaxis response regulator protein-glutamate methylesterase (EC 3.1.1.61)
SL1887	*fliB*	4.64876E-06	-6.46	Lysine-N-methylase (Ec 2.1.1.-) (Lysine N-methyltransferase)
SL2872	*invI*	4.02354E-06	-6.54	Type III secretion system secretory apparatus
SL1116	*flgG*	1.50553E-05	-6.61	Flagellar basal-body rod protein FlgG (Distal rod protein)

SL1121	*flgL*		4.64876E-06	-6.61	Flagellar hook-associated protein 3
SL2850	*orgA*		4.77054E-06	-6.72	Oxygen-regulated invasion protein OrgA
SL2860	*iacP*	*sipF*	5.45053E-06	-6.74	Probable acyl carrier protein IacP (ACP)
SL1855	*cheW*		1.82855E-05	-6.79	Purine binding chemotaxis protein
SL2863	*sipC*	*sspC*	4.02354E-06	-6.85	Cell invasion protein SipC (Effector protein SipC)
SL2864	*sipB*		4.66107E-06	-6.85	Pathogenicity island 1 Type III secretion system effector protein
SL1890	*fliS*		4.02354E-06	-6.86	Flagellar secretion chaperone FliS
SL2861	*sipA*	*sspA*	1.82855E-05	-6.89	Cell invasion protein sipA (Effector protein sipA)
SL2870	*spaO*		4.50297E-06	-6.97	Surface presentation of antigens protein (Associated with type III secretion and virulence)
SL4464	*tsr*		2.01078E-05	-7.01	Methyl-accepting chemotaxis protein
SL1029	*pipC*		9.26473E-06	-7.05	Chaperone protein
SL1556	*trg*		3.96943E-06	-7.13	Methyl-accepting chemotaxis protein III (Mcp-iii) (Ribose and galactose chemoreceptor protein)
SL1858	*motA*		4.02354E-06	-7.14	Motility protein A
SL1115	*flgF*		6.65E-06	-7.15	Flagellar basal body protein
SL2856	*hilA*		4.64876E-06	-7.17	Invasion protein regulator
SL1851	*cheY*		2.10656E-05	-7.20	Chemotaxis protein CheY
SL2756	*fljB*		1.46423E-05	-7.27	Flagellin
SL1902	*fliJ*		4.02354E-06	-7.34	Flagellar FliJ protein
SL2871	*invJ*		6.65E-06	-7.34	Surface presentation of antigens protein (Associated with type III secretion and virulence)
SL1111	*flgB*		4.64876E-06	-7.36	Flagellar basal body rod protein FlgB
SL1857	*motB*		3.71081E-06	-7.37	Motility protein B
SL2877	*invG*		4.02354E-06	-7.38	Type III secretion system secretory apparatus
SL2283			4.02354E-06	-7.40	Hypothetical receptor/regulator protein
SL2851	*prgK*		3.75238E-06	-7.55	Lipoprotein
SL2874	*invB*		4.64876E-06	-7.57	Chaperone protein for type III secretion system effectors

SL1856	*cheA*	4.64876E-06	-7.63	Chemotaxis protein CheA
SL1120	*flgK*	3.96943E-06	-7.68	Flagellar hook-associated protein 1 (HAP1)
SL1114	*flgE*	6.65E-06	-7.70	Flagellar hook protein FlgE
SL1885	*fliA*	3.31007E-06	-8.03	RNA polymerase sigma factor FliA (RNA polymerase sigma factor for flagellar operon) (Sigma F) (Sigma-28)
SL2862	*sipD*	3.44783E-06	-8.03	Pathogenicity island 1 Type III secretion system apparatus- part of the Translocon
SL2853	*prgI*	2.03828E-05	-8.08	Type III secretion system apparatus
SL1113	*flgD*	3.31007E-06	-8.19	Basal-body rod modification protein FlgD
SL3189	*0*	3.31007E-06	-8.28	Methyl-accepting chemotaxis protein II
SL1030	*sopB*	3.6878E-06	-8.29	Type III secretion system effector protein.
SL2852	*prgJ*	3.31007E-06	-8.43	Type III secretion system apparatus
SL3542	*tcp*	3.31007E-06	-8.49	Methyl-accepting chemotaxis citrate transducer
SL2878	*invF*	4.50297E-06	-8.61	AraC-family regulatory protein
SL1889	*fliD*	4.02354E-06	-8.64	Flagellar hook-associated protein 2 (HAP2) (Flagellar cap protein)
SL1112	*flgC*	6.44603E-06	-8.64	Flagellar basal-body rod protein FlgC
SL1854	*cheM*	3.31007E-06	-8.79	Methyl-accepting chemotaxis protein II
SL2674	*sopE*	3.31007E-06	-9.38	Guanine nucleotide exchange factor
