# Supplementary material for: Formation of phenotypic lineages in Salmonella enterica by a pleiotropic fimbrial switch
Source: PLoS Genet. 2018 Sep 25;14(9):e1007677. doi: 10.1371/journal.pgen.1007677 (PMC6173445; doi:10.1371/journal.pgen.1007677)
Supplement: S2 Table — (PDF) [file pgen.1007677.s002.pdf]

Table S2. Binding sites of StdE and StdF identified by ChIP-seq

StdE binding peaks						
**Location in the genome**	**Peak (start and end)**		**Map position**		**Gene description**	**DNA strand**
**pCol1B9**	250	549	455	1486	replication_initiation_protein_PK-repZ	+
**pCol1B9**	1400	1549	455	1486	replication_initiation_protein_PK-repZ	+
**pCol1B9**	8000	8199	6669	8015	hypothetical_protein	+
**pCol1B9**	30050	30199	30112	30366	hypothetical_protein	-
**pCol1B9**	86350	86599	85922	86158	conjugal_transfer_protein_TraA	-
**pRSF1010**	7200	7449	7015	7851	aminoglycoside_phosphotransferase_APH(6)-I	-
**pSLT**	29800	30149	29946	30512	conjugal_transfer_protein_TraE	-
**pSLT**	78150	78349	78078	78459	pseudo_pseudo	-
**Chromosome**	139100	139199	138340	139344	catabolite_repressor/activator	+
**Chromosome**	139750	139999	139950	140408	transcriptional_regulator_MraZ	+
**Chromosome**	174750	175049	173384	174754	aromatic_amino_acid_transporter	-
**Chromosome**	216900	216999	217026	217730	sugar_fermentation_stimulation_protein_A	-
**Chromosome**	223500	223749	223735	225924	ferrichrome_outer_membrane_transporter	+
**Chromosome**	242750	243049	242841	244358	deoxyguanosinetriphosphate_triphosphohydrolase	+
**Chromosome**	250100	250249	250139	252811	uridylyltransferase	-
**Chromosome**	288850	289049	288235	288801	D-glycero-beta-D-manno-heptose-1.7-bisphosphate_7-phosphatase	+
**Chromosome**	368150	368249	366908	368158	gamma-glutamyl_phosphate_reductase	+
**Chromosome**	386200	386499	385794	386294	hypothetical_protein	+
**Chromosome**	403450	403649	402817	404775	type_III_restriction-modification_system_StyLTI_enzyme_mod	+
**Chromosome**	413700	413849	412167	414257	ferrioxamine_B_receptor	+
**Chromosome**	414900	415049	414298	414930	transporter	-
**Chromosome**	434800	435099	435083	436294	permease	+
**Chromosome**	461150	461549	461457	461789	preprotein_translocase_subunit_YajC_yajC	+
**Chromosome**	498350	498499	498086	499561	AmpG_family_muropeptide_MFS_transporter_ampG	-
**Chromosome**	507800	508149	507591	507863	DNA-binding_protein_HU-beta	+

**Chromosome**	527450	527599	526889	527440	maltose_O-acetyltransferase	-
**Chromosome**	531850	532149	531907	533100	MexE_family_multidrug_efflux_RND_transporter_periplasmic_adaptor_subunit	-
**Chromosome**	555400	555549	555572	556366	hypothetical_protein	-
**Chromosome**	660450	660649	660167	660580	proofreading_thioesterase_EntH	+
**Chromosome**	677350	677499	677511	678749	dehydrogenase	+
**Chromosome**	692200	692399	691655	692215	phospholipid--lipid_A_palmitoyltransferase_pagP	+
**Chromosome**	701350	701699	701433	703334	penicillin-binding_protein_2	-
**Chromosome**	725900	726049	726021	726746	glutamate/aspartate_ABC_transporter_ATP-binding_protein_artP	-
**Chromosome**	738050	738349	738051	738127	tRNA-Met_tRNA-Met	-
**Chromosome**	741300	741399	741103	742323	transcriptional_regulator	-
**Chromosome**	749800	749999	748945	750351	chitoporin_chiP	+
**Chromosome**	765450	765649	765386	765499	hypothetical_protein	+
**Chromosome**	768550	769099	766424	769108	two-component_system_sensor_histidine_kinase_KdpD	-
**Chromosome**	822900	822999	823028	824080	phospho-2-dehydro-3-deoxyheptonate_aldolase	+
**Chromosome**	847500	847799	847453	848271	pyridoxal_phosphate_phosphatase	-
**Chromosome**	865300	865499	863688	865709	UvrABC_system_protein_B	+
**Chromosome**	890000	890099	889964	890104	hypothetical_protein	-
**Chromosome**	964050	964299	964160	965593	hypothetical_protein	-
**Chromosome**	965450	965649	965604	966605	threonine_aldolase	-
**Chromosome**	978700	978799	978884	979204	ATP-dependent_Clp_protease_adapter_protein_ClpS_clpS	+
**Chromosome**	1017750	1018049	1018042	1019130	phosphoserine_aminotransferase	+
**Chromosome**	1022000	1022349	1021561	1022244	cytidylate_kinase_cmk	+
**Chromosome**	1043850	1044099	1043688	1044236	outer_membrane_protein	+
**Chromosome**	1050000	1050199	1050105	1050338	hypothetical_protein	-
**Chromosome**	1086800	1087199	1086605	1087138	Superoxide_dismutase_[Cu-Zn]_1	-
**Chromosome**	1089650	1090049	1089344	1092694	host_specificity_protein_J	+
**Chromosome**	1110650	1110749	1110249	1111502	paraquat-inducible_protein_A	+
**Chromosome**	1115900	1116199	1114817	1116577	lon_protease	-
**Chromosome**	1131550	1131699	1131644	1131731	tRNA-Ser_tRNA-Ser	+
**Chromosome**	1158750	1159049	1158770	1159393	DSBA_oxidoreductase	+

**Chromosome**	1167950	1168199	1163919	1167881	bifunctional_protein_PutA_putA	-
**Chromosome**	1183500	1183699	1183644	1184126	membrane_protein	+
**Chromosome**	1192350	1192599	1192541	1194094	glucan_biosynthesis_protein_D_mdoG	+
**Chromosome**	1262600	1262749	1261987	1262898	N-acetyl-D-glucosamine_kinase	+
**Chromosome**	1289850	1290099	1290067	1290143	tRNA-Arg_tRNA-Arg	+
**Chromosome**	1306400	1306599	1306474	1306623	hypothetical_protein	-
**Chromosome**	1309750	1310099	1309991	1310239	membrane_protein	+
**Chromosome**	1326650	1326849	1326883	1327296	peptide_methionine_sulfoxide_reductase_MsrB	+
**Chromosome**	1383250	1383449	1382251	1383297	phospho-2-dehydro-3-deoxyheptonate_aldolase_Trp-sensitive	-
**Chromosome**	1399850	1400149	1399804	1401015	transporter	-
**Chromosome**	1510850	1511099	1511078	1512022	hypothetical_protein	-
**Chromosome**	1544700	1544849	1544840	1545526	hypothetical_protein	-
**Chromosome**	1550650	1550849	1550184	1550576	hypothetical_protein	+
**Chromosome**	1556950	1557149	1557061	1557933	LysR_family_transcriptional_regulator	-
**Chromosome**	1589900	1590099	1588983	1589990	transcriptional_regulator	+
**Chromosome**	1615100	1615599	1615267	1615845	TetR_family_transcriptional_regulator	+
**Chromosome**	1637850	1638049	1637948	1640929	virulence_factor_SrfB	+
**Chromosome**	1643300	1643499	1643115	1643288	hypothetical_protein	-
**Chromosome**	1658450	1658649	1658651	1659769	aminopeptidase	+
**Chromosome**	1725350	1725549	1724854	1725567	oxidoreductase	+
**Chromosome**	1743250	1743549	1743130	1744020	peptide_ABC_transporter_permease	+
**Chromosome**	1749900	1750149	1749977	1751110	hypothetical_protein	+
**Chromosome**	1764700	1764949	1764839	1764967	hypothetical_protein	-
**Chromosome**	1802000	1802349	1799981	1802659	acetaldehyde_dehydrogenase	+
**Chromosome**	1834000	1834299	1833497	1834348	4-diphosphocytidyl-2-C-methyl-D-erythritol_kinase	+
**Chromosome**	1863450	1863599	1862742	1863461	fatty_acid_metabolism_regulator_protein	-
**Chromosome**	1893450	1893699	1893337	1893480	hypothetical_protein	-
**Chromosome**	1901600	1901899	1901554	1902051	GAF_domain_protein	-
**Chromosome**	1902200	1902299	1902180	1903463	membrane_protein	+
**Chromosome**	1930600	1930799	1929373	1931424	protease	-

**Chromosome**	1935150	1935399	1935189	1937000	phosphogluconate_dehydratase	-
**Chromosome**	1972350	1972449	1972323	1973984	methyl-accepting_chemotaxis_protein_II	-
**Chromosome**	1979550	1980049	1979284	1979625	flagellar_transcriptional_regulator_FlhD	-
**Chromosome**	1980250	1980549	1980421	1980849	universal_stress_protein_C	+
**Chromosome**	1983000	1983349	1982263	1983066	trehalose-phosphate_phosphatase	-
**Chromosome**	2034450	2034549	2034188	2034454	membrane_protein	-
**Chromosome**	2039650	2039749	2039277	2039438	hypothetical_protein	-
**Chromosome**	2059350	2059549	2058604	2060337	terminase	-
**Chromosome**	2133550	2133799	2133740	2134912	D-alanyl-D-alanine_carboxypeptidase	-
**Chromosome**	2134850	2135149	2135036	2135800	thiosulfate_reductase_cytochrome_B	-
**Chromosome**	2139050	2139199	2139285	2141633	E3_ubiquitin--protein_ligase	+
**Chromosome**	2154000	2154199	2153855	2154466	histidine_biosynthesis_bifunctional_protein_HisIE	+
**Chromosome**	2177050	2177249	2176672	2177565	UTP--glucose-1-phosphate_uridylyltransferase	-
**Chromosome**	2234750	2234899	2233987	2235258	nucleoside_permease	+
**Chromosome**	2255700	2255849	2255678	2256625	ABC_transporter_ATP-binding_protein	-
**Chromosome**	2264450	2264699	2264354	2264941	transporter	-
**Chromosome**	2266300	2266599	2266545	2267981	lipoprotein	-
**Chromosome**	2276650	2276949	2276882	2277766	cytidine_deaminase	+
**Chromosome**	2286850	2287149	2287003	2288145	membrane_protein	-
**Chromosome**	2311200	2311299	2311374	2311946	membrane_protein_spr	+
**Chromosome**	2317850	2318149	2317871	2319460	microcin_C_ABC_transporter_ATP-binding_protein_YejF	+
**Chromosome**	2332500	2332899	2332550	2333077	tail_protein	-
**Chromosome**	2357150	2357349	2356870	2357361	WP_000228070.1_ferredoxin	-
**Chromosome**	2406050	2406249	2405788	2406687	4-deoxy-4-formamido-L-arabinose-phosphoundecaprenol_deformylase_ArnD	+
**Chromosome**	2412100	2412249	2411715	2412572	1.4-dihydroxy-2-naphthoyl-CoA_synthase	-
**Chromosome**	2450300	2450599	2449296	2450816	hypothetical_protein	+
**Chromosome**	2475150	2475449	2475070	2476338	bifunctional_folylpolyglutamate_synthase/_dihydrofolate_synthase	-
**Chromosome**	2499800	2499949	2499456	2499740	hypothetical_protein	-
**Chromosome**	2508800	2509249	2508217	2509491	ABC_transporter_substrate-binding_protein	-
**Chromosome**	2509400	2509549	2509454	2509672	hypothetical_protein	-

**Chromosome**	2522750	2523149	2523019	2524221	WP_000376347.1_nucleoside_permease	+
**Chromosome**	2526800	2526949	2526772	2526847	tRNA-Ala_tRNA-Ala	-
**Chromosome**	2541900	2542049	2542037	2542162	hypothetical_protein	-
**Chromosome**	2582450	2582599	2581216	2583216	transketolase	+
**Chromosome**	2595600	2595999	2595410	2596537	succinyl-diaminopimelate_desuccinylase	+
**Chromosome**	2599500	2599599	2598826	2599689	hypothetical_protein	-
**Chromosome**	2610200	2610399	2609749	2610375	uracil_phosphoribosyltransferase_upp	-
**Chromosome**	2623650	2623849	2623795	2625144	exodeoxyribonuclease_7_large_subunit_xseA	+
**Chromosome**	2647300	2647549	2646325	2648517	intimin	-
**Chromosome**	2661250	2661649	2660629	2663007	DMSO_reductase_subunit_A	-
**Chromosome**	2706400	2706599	2705971	2709858	phosphoribosylformylglycinamidine_synthase	-
**Chromosome**	2718850	2719049	2718443	2719369	LysR_family_transcriptional_regulator	+
**Chromosome**	2790150	2790299	2790293	2792953	protein_lysine_acetyltransferase_Pat	+
**Chromosome**	2802400	2802599	2802529	2805102	chaperone_protein_ClpB	-
**Chromosome**	2807850	2808099	2808081	2808419	translation_inhibitor_protein_RaiA	+
**Chromosome**	2821400	2821699	2820345	2822183	partial;pseudo	+
**Chromosome**	2887950	2888199	2887497	2888522	recombinase	+
**Chromosome**	2935050	2935349	2935199	2935738	repressor_of_phase_1_flagellin_gene	-
**Chromosome**	2956900	2957049	2956337	2957011	two-component_system_response_regulator	-
**Chromosome**	2973900	2974099	2973730	2974161	alkylhydroperoxidase	+
**Chromosome**	3041050	3041199	3040412	3041341	transcriptional_regulator_hilD	+
**Chromosome**	3050500	3050599	3050433	3051662	cell_invasion_protein_SipC	-
**Chromosome**	3084300	3084549	3084385	3085149	DeoR_family_transcriptional_regulator	+
**Chromosome**	3089500	3090249	3089110	3090243	murein_hydrolase_activator_NlpD_nlpD	-
**Chromosome**	3137150	3137399	3137007	3137789	tRNA_pseudouridine_synthase_C	-
**Chromosome**	3138250	3138399	3138274	3138486	hypothetical_protein	-
**Chromosome**	3156400	3156699	3155734	3156651	transcriptional_regulator	-
**Chromosome**	3162300	3162499	3161221	3162318	murein_transglycosylase_mltA	-
**Chromosome**	3166600	3166849	3165738	3167573	exonuclease_V_subunit_alpha_recD	-
**Chromosome**	3181100	3181199	3180280	3181155	prolipoprotein_diacylglyceryl_transferase	-

**Chromosome**	3184350	3184549	3184511	3184657	hypothetical_protein		-
**Chromosome**	3205600	3205899	3205639	3206475	cobalt_transporter	yohM	+
**Chromosome**	3215600	3215899	3215876	3216274	tRNA(fMet)-specific_endonuclease_VapC		-
**Chromosome**	3227500	3227599	3227387	3227623	hypothetical_protein		+
**Chromosome**	3242800	3243049	3243022	3243618	5-formyltetrahydrofolate_cyclo-ligase		+
**Chromosome**	3247250	3247349	3247215	3247466	hypothetical_protein		+
**Chromosome**	3253700	3253949	3252597	3253643	D-erythrose-4-phosphate_dehydrogenase_gapA		-
**Chromosome**	3259700	3260099	3259380	3260138	metalloprotease		+
**Chromosome**	3330950	3331099	3329490	3331193	hydrogenase_2_large_subunit		-
**Chromosome**	3350700	3351049	3349642	3351813	radical_SAM_protein		-
**Chromosome**	3383050	3383249	3383264	3384697	bifunctional_protein_HldE		-
**Chromosome**	3385050	3385549	3384745	3387588	glutamate--ammonia-ligase_adenylyltransferase		-
**Chromosome**	3394450	3394849	3394760	3394975	30S_ribosomal_protein_S21_rpsU		+
**Chromosome**	3399550	3399849	3399709	3399784	tRNA-Met_tRNA-Met		+
**Chromosome**	3409000	3409249	3409059	3410195	ribosomal_RNA_large_subunit_methyltransferase_G		-
**Chromosome**	3435850	3435999	3435915	3437060	glycerate_kinase		-
**Chromosome**	3447250	3447649	3447534	3448805	tagatose-1.6-bisphosphate_aldolase		+
**Chromosome**	3452000	3452249	3452174	3452947	galactitol_utilization_operon_repressor		+
**Chromosome**	3468800	3468949	3468958	3469842	lipoprotein_NlpI		-
**Chromosome**	3479200	3479549	3479208	3479284	tRNA-Met_tRNA-Met		-
**Chromosome**	3522550	3522699	3522035	3523138	hypothetical_protein		+
**Chromosome**	3530400	3530699	3530564	3531355	transcriptional_regulator_NanR		-
**Chromosome**	3578500	3578649	3578064	3578948	methyltransferase		+
**Chromosome**	3597750	3598299	3597162	3598286	hypothetical_protein		-
**Chromosome**	3618150	3618299	3618392	3618859	leader_peptidase_HopD		+
**Chromosome**	3636800	3637049	3636416	3636820	hypothetical_protein		-
**Chromosome**	3643350	3643549	3643554	3644735	protein_TsgA		+
**Chromosome**	3664300	3664499	3663746	3664267	shikimate_kinase_aroK		-
**Chromosome**	3671300	3671549	3670820	3671383	adenosine_nucleotide_hydrolase_nudE		-
**Chromosome**	3677700	3678099	3678085	3679704	phosphoenolpyruvate_carboxykinase_[ATP]		+

**Chromosome**	3714950	3715149	3713815	3714924	glycerol_dehydrogenase_gldA	-
**Chromosome**	3736800	3736899	3736939	3737427	acetyltransferase	+
**Chromosome**	3749800	3749949	3749623	3749844	prevent-host-death_family_protein	-
**Chromosome**	3758150	3758299	3757249	3758103	RNA_polymerase_sigma-32_factor	-
**Chromosome**	3796650	3796899	3796899	3798548	trehalase_treF	+
**Chromosome**	3804100	3804349	3804377	3805699	transporter	+
**Chromosome**	3829500	3829749	3829506	3829613	hypothetical_protein	-
**Chromosome**	3841750	3841949	3841901	3843592	phosphoethanolamine_transferase_EptB	-
**Chromosome**	3871650	3871749	3871475	3871846	hypothetical_protein	+
**Chromosome**	3896100	3896599	3896582	3898498	PTS_mannitol_transporter_subunit_IIABC	+
**Chromosome**	3902950	3903349	3902543	3906928	membrane_protein	+
**Chromosome**	3912600	3912749	3912770	3913966	L-talarate/galactarate_dehydratase	+
**Chromosome**	3921850	3922099	3921719	3922681	hypothetical_protein	+
**Chromosome**	3945900	3946249	3946033	3946491	deoxyuridine_5'-triphosphate_nucleotidohydrolase	+
**Chromosome**	3951700	3951949	3951856	3952473	membrane_protein	+
**Chromosome**	3957300	3957549	3957500	3958189	tRNA_methyltransferase	+
**Chromosome**	3999850	4000049	3998699	3999891	pseudo;old_locus_tag=CHROMOSOME_3741_	-
**Chromosome**	4006550	4006699	4005752	4006495	GntR_family_transcriptional_regulator	-
**Chromosome**	4019100	4019349	4019024	4019122	ilvB_operon_leader_peptide	-
**Chromosome**	4045350	4045949	4044666	4045850	trimethylamine_N-oxide_reductase_cytochrome_C_subunit	-
**Chromosome**	4058750	4058949	4057949	4059139	MR-MLE_family_protein	+
**Chromosome**	4066800	4066899	4066999	4067139	50S_ribosomal_protein_L34_rpmH	+
**Chromosome**	4101950	4102249	4101487	4101867	F0F1_ATP_synthase_subunit_I	-
**Chromosome**	4127900	4128049	4128045	4128383	hypothetical_protein hdfR	+
**Chromosome**	4146750	4147049	4146381	4146710	thioredoxin_trxA	+
**Chromosome**	4196250	4196399	4196409	4197170	uridine_phosphorylase	+
**Chromosome**	4227150	4227549	4226273	4227181	acyltransferase	-
**Chromosome**	4230250	4230449	4227569	4230355	DNA_polymerase_I	+
**Chromosome**	4238550	4238899	4238822	4240645	GTP-binding_protein	+
**Chromosome**	4244100	4244199	4243537	4244412	membrane_protein	-

**Chromosome**	4252950	4253299	4253044	4254285	sulfoquinovose_isomerase	-
**Chromosome**	4280300	4280449	4280503	4281339	transcriptional_activator_RhaS	+
**Chromosome**	4283500	4283799	4283840	4283965	hypothetical_protein	+
**Chromosome**	4284750	4284999	4284831	4286138	membrane_protein	-
**Chromosome**	4348350	4348749	4348739	4349818	PTS_fructose_transporter_subunit_IIC	+
**Chromosome**	4384200	4384399	4383733	4384278	transcription_termination/antitermination_protein_NusG_nusG	+
**Chromosome**	4407800	4408049	4407263	4407853	hypothetical_protein	+
**Chromosome**	4415700	4415949	4416006	4417559	16S_ribosomal_RNA_16S_ribosomal_RNA	+
**Chromosome**	4471750	4472149	4470620	4471810	maltose-binding_periplasmic_protein_malE	-
**Chromosome**	4479250	4479499	4477472	4479892	glycerol-3-phosphate_acyltransferase	-
**Chromosome**	4523150	4523349	4522829	4523110	membrane_protein	-
**Chromosome**	4537050	4537149	4537151	4537297	entericidin_B_precursor	+
**Chromosome**	4544800	4545049	4544308	4545618	proton_glutamate_symport_protein	+
**Chromosome**	4572450	4572599	4571185	4572816	sensor_histidine_kinase	-
**Chromosome**	4593100	4593249	4592995	4593117	hypothetical_protein	-
**Chromosome**	4594850	4595199	4593838	4595079	transporter	-
**Chromosome**	4639450	4639599	4638946	4640109	glutathionylspermidine_synthase	+
**Chromosome**	4642450	4642649	4642267	4642596	transporter	-
**Chromosome**	4650900	4650999	4650793	4651023	hypothetical_protein	+
**Chromosome**	4651600	4651699	4651496	4651810	primosomal_replication_protein_n	+
**Chromosome**	4655200	4655299	4654512	4655132	peptidyl-prolyl_cis-trans_isomerase	+
**Chromosome**	4704250	4704549	4704533	4704862	glycine_dehydrogenase	+
**Chromosome**	4810450	4810599	4810665	4812326	methyl-accepting_chemotaxis_protein	+
**Chromosome**	4836200	4836449	4836462	4837079	hypothetical_protein	+
**Chromosome**	4838900	4839199	4838563	4839336	deoxyribonuclease	+
**Chromosome**	4841750	4841999	4840204	4841751	hypothetical_protein	-
**StdF binding peaks**						
**pCol1B9**	1400	1499	455	1486	replication_initiation_protein_PK-repZ	+
**pCol1B9**	1600	1799	455	1486	replication_initiation_protein_PK-repZ	+

**pCol1B9**	32350	32599	32467	33315	hypothetical_protein	+
**pCol1B9**	33250	33499	32467	33315	hypothetical_protein	+
**pCol1B9**	34300	34549	34396	37011	hypothetical_protein	+
**pCol1B9**	72300	72549	71003	72295	Shufflon_protein_A'	-
**pCol1B9**	75000	75349	75248	76801	ATP-binding_protein	-
**pSLT**	12550	12899	11189	14011	conjugal_transfer_protein_TraG	-
**pSLT**	29750	30149	29946	30512	conjugal_transfer_protein_TraE	-
**pSLT**	30250	30349	29946	30512	conjugal_transfer_protein_TraE	-
**pSLT**	41300	41449	41218	41652	hypothetical_protein	-
**pSLT**	61000	61099	59874	60911	integrase	-
**pSLT**	71500	71699	71594	72376	resolvase	-
**pSLT**	78750	78849	78078	78459	pseudo_pseudo	-
**Chromosome**	23050	23299	20058	23054	chitinase	+
**Chromosome**	242850	242999	242841	244358	deoxyguanosinetriphosphate_triphosphohydrolase	+
**Chromosome**	256900	257249	257144	257701	ribosome-recycling_factor_frr	+
**Chromosome**	258950	259149	257842	259038	1-deoxy-D-xylulose_5-phosphate_reductoisomerase	+
**Chromosome**	388200	388299	388268	389011	transcriptional_regulator	+
**Chromosome**	413800	413899	412167	414257	ferrioxamine_B_receptor	+
**Chromosome**	492150	492249	491265	492758	hypothetical_protein	+
**Chromosome**	531350	531499	528735	531884	multidrug_efflux_RND_transporter_permease_subunit	-
**Chromosome**	660500	660599	660167	660580	proofreading_thioesterase_EntH	+
**Chromosome**	701350	701649	701433	703334	penicillin-binding_protein_2	-
**Chromosome**	741000	741149	741103	742323	transcriptional_regulator	-
**Chromosome**	749400	749649	748945	750351	chitoporin_chiP	+
**Chromosome**	768550	769199	769105	769689	potassium-transporting_ATPase_C_chain	-
**Chromosome**	822850	822949	823028	824080	phospho-2-dehydro-3-deoxyheptonate_aldolase	+
**Chromosome**	840150	840499	839835	840851	UDP-glucose_4-epimerase	-
**Chromosome**	847450	847849	847453	848271	pyridoxal_phosphate_phosphatase	-

**Chromosome**	854950	855199	854223	855908	urocanate_hydratase	+
**Chromosome**	861150	861299	860462	861619	8-amino-7-oxononanoate_synthase	+
**Chromosome**	873350	873499	873495	874202	membrane_protein	+
**Chromosome**	878950	879049	878868	879974	ABC_transporter_permease	-
**Chromosome**	898550	898699	898795	899310	outer_membrane_protease_ompX	+
**Chromosome**	901650	901849	901532	902005	transcriptional_regulator_MntR	+
**Chromosome**	965400	965699	965604	966605	threonine_aldolase	-
**Chromosome**	1033850	1034149	1033973	1034776	S-adenosyl-L-methionine-dependent_methyltransferase	+
**Chromosome**	1043900	1044049	1043688	1044236	outer_membrane_protein	+
**Chromosome**	1086550	1086799	1086605	1087138	Superoxide_dismutase_[Cu-Zn]_1	-
**Chromosome**	1087100	1087249	1087228	1087923	tail_protein	+
**Chromosome**	1089750	1090049	1089344	1092694	host_specificity_protein_J	+
**Chromosome**	1096900	1097149	1096293	1097261	secreted_effector_protein_SseI	+
**Chromosome**	1099700	1099949	1099845	1100036	DNA-damage-inducible_protein_I	-
**Chromosome**	1112550	1112849	1111507	1113147	paraquat-inducible_protein_B	+
**Chromosome**	1115850	1116149	1114817	1116577	lon_protease	-
**Chromosome**	1135700	1135999	1134553	1136238	inositol_phosphate_phosphatase_SopB_sopB	-
**Chromosome**	1158850	1158999	1158770	1159393	DSBA_oxidoreductase	+
**Chromosome**	1172750	1173049	1172764	1174260	acetylneuraminate_ABC_transporter	-
**Chromosome**	1189450	1189799	1189629	1191116	hypothetical_protein	+
**Chromosome**	1269900	1270149	1269559	1270788	peptidase_T	+
**Chromosome**	1289850	1290049	1289672	1289843	pseudo_pseudo	-
**Chromosome**	1290200	1290499	1290067	1290143	tRNA-Arg_tRNA-Arg	+
**Chromosome**	1327050	1327199	1326883	1327296	peptide_methionine_sulfoxide_reductase_MsrB	+
**Chromosome**	1446800	1446949	1446884	1447132	type_III_secretion_system_protein_SsaI	+
**Chromosome**	1484900	1485099	1483302	1484807	dipeptide_and_tripeptide_permease_A_tppB	-
**Chromosome**	1550850	1551099	1550924	1551019	membrane_protein	-
**Chromosome**	1601800	1602049	1602034	1602249	protein_bdm	+

**Chromosome**	1665600	1665799	1664695	1665897	L-lactate_oxidase	-
**Chromosome**	1734250	1734599	1733034	1734575	transcriptional_regulator	-
**Chromosome**	1787550	1787699	1787652	1788053	acyl-CoA_thioester_hydrolase_YciA	+
**Chromosome**	1894600	1895149	1894788	1895027	hypothetical_protein	+
**Chromosome**	1901650	1901799	1901554	1902051	GAF_domain_protein	-
**Chromosome**	1902050	1902349	1902180	1903463	membrane_protein	+
**Chromosome**	1904450	1904549	1903432	1906065	hypothetical_protein	+
**Chromosome**	1927300	1927449	1927110	1927484	hypothetical_protein	-
**Chromosome**	1935000	1935399	1935189	1937000	phosphogluconate_dehydratase	-
**Chromosome**	1972350	1972499	1972323	1973984	methyl-accepting_chemotaxis_protein_II	-
**Chromosome**	1979550	1979999	1979284	1979625	flagellar_transcriptional_regulator_FlhD	-
**Chromosome**	1980300	1980749	1980421	1980849	universal_stress_protein_C	+
**Chromosome**	1995400	1995649	1993986	1995818	UvrABC_system_protein_C_uvrC	-
**Chromosome**	2072150	2072299	2072244	2072939	transcriptional_regulator	+
**Chromosome**	2140300	2140399	2139285	2141633	E3_ubiquitin--protein_ligase	+
**Chromosome**	2187750	2187849	2187655	2188878	glycosyl_transferase	-
**Chromosome**	2264000	2264299	2263241	2264188	D-alanyl-D-alanine_endopeptidase_pbpG	-
**Chromosome**	2291900	2292099	2291982	2292869	phosphoserine_phosphatase	+
**Chromosome**	2292700	2292949	2292902	2294224	hypothetical_protein	+
**Chromosome**	2317900	2317999	2317871	2319460	microcin_C_ABC_transporter_ATP-binding_protein_YejF	+
**Chromosome**	2332500	2332899	2332550	2333077	tail_protein	-
**Chromosome**	2335700	2335999	2335540	2336871	NTPase	+
**Chromosome**	2442500	2442649	2441710	2443536	transcriptional_regulator	-
**Chromosome**	2450300	2450499	2449296	2450816	hypothetical_protein	+
**Chromosome**	2475300	2475449	2475070	2476338	bifunctional_folylpolyglutamate_synthase/_dihydrofolate_synthase	-
**Chromosome**	2508850	2509249	2508217	2509491	ABC_transporter_substrate-binding_protein	-
**Chromosome**	2509350	2509649	2509454	2509672	hypothetical_protein	-
**Chromosome**	2595650	2596099	2595410	2596537	succinyl-diaminopimelate_desuccinylase	+

**Chromosome**	2610750	2610899	2610789	2611826	phosphoribosylformylglycinamidine_cyclo-ligase	+
**Chromosome**	2633050	2633199	2632119	2639425	pseudo_pseudo	-
**Chromosome**	2656550	2656799	2656413	2657579	23S_rRNA_(adenine(2503)-C(2))-methyltransferase_RlmN	-
**Chromosome**	2661200	2661549	2660629	2663007	DMSO_reductase_subunit_A	-
**Chromosome**	2680950	2681049	2680648	2681142	Fe-S_cluster_assembly_transcriptional_regulator_IscR	-
**Chromosome**	2681950	2682199	2682152	2682955	inositol-1-monophosphatase	+
**Chromosome**	2684350	2684699	2684160	2685203	sulfite_reductase_subunit_alpha	+
**Chromosome**	2695950	2696199	2696253	2697584	arginine:agmatine_antiporter_cadB	+
**Chromosome**	2706450	2706649	2705971	2709858	phosphoribosylformylglycinamidine_synthase	-
**Chromosome**	2726800	2727049	2726717	2728186	type_III_secretion_system_protein	+
**Chromosome**	2728050	2728199	2726717	2728186	type_III_secretion_system_protein	+
**Chromosome**	2821300	2821599	2820345	2822183	partial;pseudo_partial;pseudo	+
**Chromosome**	2887150	2887349	2886863	2887495	phage_repressor_protein	+
**Chromosome**	2888850	2888949	2888519	2889730	hypothetical_protein	+
**Chromosome**	2929950	2930199	2929642	2929908	transposase	-
**Chromosome**	2951200	2951399	2950949	2951878	VirG_localization_protein_VirK	+
**Chromosome**	3027750	3027999	3026281	3028359	formate_hydrogenlyase_transcriptional_activator	+
**Chromosome**	3051500	3051699	3051690	3053471	cell_invasion_protein_SipB	-
**Chromosome**	3089550	3090049	3089110	3090243	murein_hydrolase_activator_NlpD_nlpD	-
**Chromosome**	3117500	3117749	3118020	3118691	7-carboxy-7-deazaguanine_synthase	-
**Chromosome**	3165650	3165799	3165738	3167573	exonuclease_V_subunit_alpha_recD	-
**Chromosome**	3181050	3181149	3180280	3181155	prolipoprotein_diacylglyceryl_transferase	-
**Chromosome**	3205450	3205999	3205639	3206475	cobalt_transporter yohM	+
**Chromosome**	3213200	3213649	3212552	3213136	fimbrial_protein_StdA	-
**Chromosome**	3214050	3214299	3213855	3214487	hypothetical_protein	-
**Chromosome**	3259700	3260149	3259380	3260138	metalloprotease	+
**Chromosome**	3338700	3338949	3338755	3339159	hypothetical_protein	+
**Chromosome**	3369000	3369149	3368438	3369070	ADP-ribose_pyrophosphatase_nudF	-

**Chromosome**	3385350	3385549	3384745	3387588	glutamate--ammonia-ligase_adenylyltransferase	-
**Chromosome**	3504100	3504199	3503949	3504524	LPS_export_ABC_transporter_periplasmic_protein_LptC	+
**Chromosome**	3522450	3522799	3522035	3523138	hypothetical_protein	+
**Chromosome**	3552500	3552849	3551420	3553387	p-hydroxybenzoic_acid_efflux_pump_subunit_AaeB	-
**Chromosome**	3578400	3578749	3578064	3578948	methyltransferase	+
**Chromosome**	3597600	3598349	3597162	3598286	hypothetical_protein	-
**Chromosome**	3643950	3644049	3643554	3644735	protein_TsgA	+
**Chromosome**	3677750	3678149	3678085	3679704	phosphoenolpyruvate_carboxykinase_[ATP]	+
**Chromosome**	3678600	3678749	3678085	3679704	phosphoenolpyruvate_carboxykinase_[ATP]	+
**Chromosome**	3729500	3729649	3727417	3729603	1.4-alpha-glucan-branching_protein	-
**Chromosome**	3743550	3743899	3743652	3744068	outer_membrane_protein	+
**Chromosome**	3768700	3768999	3768246	3768911	membrane_protein	+
**Chromosome**	3782200	3782349	3782177	3782611	universal_stress_protein_A	+
**Chromosome**	3802600	3802799	3802026	3802925	LysR_family_transcriptional_regulator	+
**Chromosome**	3853100	3853449	3851635	3853968	biotin_sulfoxide_reductase	-
**Chromosome**	3873800	3873999	3871846	3873873	alpha-amylase_malS	+
**Chromosome**	3903000	3903299	3902543	3906928	membrane_protein	+
**Chromosome**	3949800	3949999	3949748	3950650	LysR_family_transcriptional_regulator	-
**Chromosome**	3957250	3957399	3955384	3957495	bifunctional_(p)ppGpp_synthetase_II/_guanosine-3'.5'-bis_pyrophosphate_3'-pyrophosphohydrolase	-
**Chromosome**	3962400	3962649	3962566	3963957	xanthine_permease	+
**Chromosome**	3974900	3975049	3975383	3976000	hypothetical_protein	+
**Chromosome**	4008950	4009049	4009026	4010354	regulatory_protein_UhpC	-
**Chromosome**	4020900	4021099	4021002	4022186	multidrug_resistance_protein_D_emrD	+
**Chromosome**	4028400	4028549	4028049	4028396	membrane_protein	-
**Chromosome**	4045300	4045899	4044666	4045850	trimethylamine_N-oxide_reductase_cytochrome_C_subunit	-
**Chromosome**	4046100	4046299	4045980	4046672	two-component_system_response_regulator	+
**Chromosome**	4167550	4167849	4166594	4167535	porphobilinogen_deaminase_hemC	-

**Chromosome**	4227200	4227649	4227569	4230355	DNA_polymerase_I	+
**Chromosome**	4250650	4250849	4250103	4252139	alpha-glucosidase	-
**Chromosome**	4252650	4252799	4252183	4253040	aldose-1-epimerase	-
**Chromosome**	4252900	4253249	4253044	4254285	sulfoquinovose_isomerase	-
**Chromosome**	4253750	4253949	4253044	4254285	sulfoquinovose_isomerase	-
**Chromosome**	4283550	4283799	4283840	4283965	hypothetical_protein	+
**Chromosome**	4284700	4285049	4284831	4286138	membrane_protein	-
**Chromosome**	4317100	4317199	4315780	4317288	glycerol_kinase_glpK	-
**Chromosome**	4320600	4320799	4320588	4321919	ATP-dependent_protease_ATPase_subunit_HslU_hslU	-
**Chromosome**	4323450	4323699	4323619	4324644	transcriptional_regulator	-
**Chromosome**	4336050	4336299	4335722	4336720	membrane_protein	-
**Chromosome**	4354750	4354849	4354747	4356480	phosphoethanolamine_transferase_CptA	-
**Chromosome**	4407500	4407949	4407263	4407853	hypothetical_protein	+
**Chromosome**	4408950	4409049	4408324	4409016	membrane_protein	+
**Chromosome**	4474150	4474299	4473370	4474728	maltoporin_lamB	+
**Chromosome**	4501900	4501999	4501156	4502475	ABC_transporter	+
**Chromosome**	4519450	4519599	4503766	4520445	membrane_protein	+
**Chromosome**	4544950	4545199	4544308	4545618	proton_glutamate_symport_protein	+
**Chromosome**	4582550	4582799	4582602	4582823	hypothetical_protein	-
**Chromosome**	4595000	4595349	4593838	4595079	transporter	-
**Chromosome**	4607550	4607749	4606676	4607653	elongation_factor_P--(R)-beta-lysine_ligase	+
**Chromosome**	4762500	4762599	4761955	4762767	hypothetical_protein	-
**Chromosome**	4791150	4791299	4791267	4792538	membrane_protein	-
**Chromosome**	4838600	4838949	4838563	4839336	deoxyribonuclease	+
