# Supplementary material for: Formation of phenotypic lineages in Salmonella enterica by a pleiotropic fimbrial switch
Source: PLoS Genet. 2018 Sep 25;14(9):e1007677. doi: 10.1371/journal.pgen.1007677 (PMC6173445; doi:10.1371/journal.pgen.1007677)
Supplement: S3 Table — (PDF) [file pgen.1007677.s003.pdf]

Table S3. Strains of *Salmonella enterica* used in this study

Strain	Genotype	Reference[a]
**SL1344**	Wild type	Hoiseth & Stocker. 1981
**JH3294**	Δ*dam*-231	Balbontín et al.. 2006
**SV9597**	*stdA::gfp*	
**SV9598**	Δ*hdfR* *stdA::gfp*	
**SV8141**	P_{LtetO}-*stdEF* (Cm^R)	
**SV8142**	P_{LtetO}-Δ*stdEF* (Cm^R)	
**SV7553**	P_{LtetO}-*stdEF* (Km^R)	
**SV7552**	P_{LtetO}-Δ*stdEF* (Km^R)	
**SV9324**	Δ*dam231* *stdE*::3xFLAG	
**SV9325**	Δ*dam231* *stdF*::3xFLAG	
**SV9287**	P_{LtetO}-*stdEF*::3xFLAG	
**SV9288**	*flhC::lacZ*	Laboratory stock
**SV9289**	P_{LtetO}-*stdEF flhC::lacZ*	
**SV9290**	P_{LtetO}-Δ*stdEF flhC::lacZ*	
**SV5197**	*fliC*::MudJ	Balbontín et al.. 2006
**SV8104**	P_{LtetO}-*stdEF fliC*::MudJ	
**SV8105**	P_{LtetO}-Δ*stdEF fliC*::MudJ	
**SV8106**	Δ*flhC fliC*::MudJ	
**SV8107**	P_{LtetO}-*stdEF* Δ*flhC* *fliC*::MudJ	
**SV8108**	P_{LtetO}-Δ*stdEF* Δ*flhC fliC*::MudJ	
**SV9109**	P_{LtetO}-*stdEF* Δ*flhC*	
**SV9110**	P_{LtetO}-Δ*stdEF* Δ*flhC*	
**SV7884**	*sipB::gfp*	Laboratory stock
**SV7885**	P_{LtetO}-*stdEF sipB::gfp*	
**SV7886**	P_{LtetO}-Δ*stdEF sipB::gfp*	

**SV9244**	ΔSPI-1	Laboratory stock
**SV4938**	*trg::mudQ* pSLT^{-}	García-Quintanilla & Casadesús. 2011
**SV7556**	*spvA::tn5dKm*	García-Quintanilla & Casadesús. 2011
**SV7554**	P_{LtetO}-*stdEF spvA*	
**SV7555**	P_{LtetO}-Δ*stdEF spvA*	
**SV7551**	*traB::lacZ*	Laboratory stock
**SV7550**	P_{LtetO}-*stdEF traB::lacZ*	
**SV7549**	P_{LtetO}-Δ*stdEF traB::lacZ*	
**SV8152**	Δ*ygiD*	
**SV9600**	*stdA*::3xFLAG	
**pIZ1991**	BL-21 pET28a-*stdE*	

[a] Omitted for strains constructed for this study

References

Balbontín R. Rowley G. Pucciarelli G. García del Portillo F. Hinton JCD. Casadesús J (2006). Identification of virulence genes regulated by DNA adenine methylation in *Salmonella enterica* serovar Typhimurium. Journal of Bacteriology 188: 8160-8168

García-Quintanilla M. Casadesús J (2011) Virulence plasmid interchange between strains ATCC 14028. LT2. and SL1344 of *Salmonella enterica* serovar Typhimurium. Plasmid 65: 169-17

Hoiseth SK. Stocker BA (1981) Aromatic-dependent *Salmonella typhimurium* are non-virulent and effective as live vaccines. Nature 291: 238-239
