# Supplementary material for: Formation of phenotypic lineages in Salmonella enterica by a pleiotropic fimbrial switch
Source: PLoS Genet. 2018 Sep 25;14(9):e1007677. doi: 10.1371/journal.pgen.1007677 (PMC6173445; doi:10.1371/journal.pgen.1007677)
Supplement: S4 Table — (PDF) [file pgen.1007677.s004.pdf]

Table S4. Oligonucleotides used in this study

NAME	SEQUENCE (5'→3')
stdASTOP GFP P1	TGTTAACGCATCCGTCACCTACACCCTGAAATACCTGTGATAAGAAGGAGATATACATATG
stdAGFP P2	CCTCCGTGGACGGCTTCTCCCTGTCGTTATTTACCGCGTGTTATCACTTATTCAGGCGTA
stdA E1	GGAAAGTTCAGGTGCTTCG
stdA E2	GCTTTCGGTGTTGTCGTCC
Cm-P1-R	TTCAGGCGTAGCACCAGGCGTTTAAGGGCACCAATAACTGTGTAGGCTGGAGCTGCTTCG
FliC-GFP-Km-P4	TGCTGGGATTACACATGGCATGGATGAGCTCTACAAATAAATTCCGGGGATCCGTCGACC
Km PLtetO UP	TTAATGTCATGATAATAATGGTTTCTTAGACGTCGATATCGTGTAGGCTGGAGCTGCTTC
Km PLtetO DO	CTTGGATTCTCACCAATAAAAAACGCCCGGCGGCAACCGACATATGAATATCCTCCTTAG
NdeIstdE-FOR	TTTTCATATGTGCCCTGATAATACACA
EcoRIstdE-REV	TTTTGAATTCTTATGTTCCGTCATCCTCATCC
ygiD UP	GGTTATCTGAGTTCTTCCTCTGTGAAGAAAGCGTATGGTGGTGTAGGCTGGAGCTGCTTC
ygiD DO	CGCCATCGGGCAATCATTCAGCGCCTTATCCGGCCTACCCCATATGAATATCCTCCTTAG
ygiD E1	TATCAATTTGCCGTCCGAACC
ygiD E2	TCGAAGGATAGATTCTCCTG
flhC P1	CATGATAATGAGTGAAAAAAGCATTGTTCAGGAAGCTCGCGGTGTAGGCTGGAGCTGCC
flhC P2	TTAAACAGCCTGTTCGATCTGTTCATCCAGCAGTTGTGGACATATGAATATCCTCCTTAG
flhC E1	CAATGAAGTGGACGATACGG
flhC E2	GTAATGACTTACCGCTGCTG
RT-flgE DIR	GACGGTACGACAACGAACAC
RT-flgE REV	AAAACCGTTCTGGCTAATCG
RT-hilA DIR	GAATCTTTTCATGGCTGGTCA
RT-hilA REV	GGGTCCAATTTTAAACACTCGT
RT-motA DIR	TAGGGGCGTTCATTGTCG
RT-motA REV	ACGGGATAGCTTTCATCGTG
RT-sipB DIR	GTAATGGTGGCCGATGAAAT
RT-sipB REV	CGCCTGCTGAATAAACGAC
RT-traA DIR	GTACGGTCAAGGCGACATTT

RT-traA REV	CCGCGAGAATAACCCACTT
RT-trg DIR	CGGGGTCGTACAAACGAT
RT-trg REV	GCGGTAATTTCCGAGATTTTT
RT-hdfR DIR	TTGGCGTTATATTGCAGCAG
RT-hdfR REV	CCAGCCGCAGATAATTGAGT
RT-cheA DIR	CGGTGATGTCGATTCGTATG
RT-cheAREV	TCTACCTGCTTGCCCAGTTT
RT-cheM DIR	GAAGGTTCGGATGCGATTTA
RT-cheM REV	ACGGGAAGAGAGGTCGGTAT
RT-cheB DIR	GCTGCTCAGTTCGGAAAAAC
RT-cheB REV	GCACATGTCGGATAGCTTCA
RT-stdA DIR	CATCACCAACTCACCCTGTG
RT-stdA REV	AAATCTGTCCAAACGGAACG
RT-rfaH DIR	TCAGCCATTTTGTGCGCTT
RT-rfaH REV	TTTAGGATCGACAACGCCTT
stdA 3xflag UP	CGATGTTAACGCATCCGTCACCTACACCCTGAAATACCTGGACTACAAAGACCATGACGG
stdA 3xflag DO	CCGTGGACGGCTTCTCCCTGTCGTTATTTACCGCGTGAAACATATGAATATCCTCCTTAG
