# Supplementary material for: Formation of phenotypic lineages in Salmonella enterica by a pleiotropic fimbrial switch
Source: PLoS Genet. 2018 Sep 25;14(9):e1007677. doi: 10.1371/journal.pgen.1007677 (PMC6173445; doi:10.1371/journal.pgen.1007677)
Supplement: S1 Fig — (PDF) [file pgen.1007677.s005.pdf]

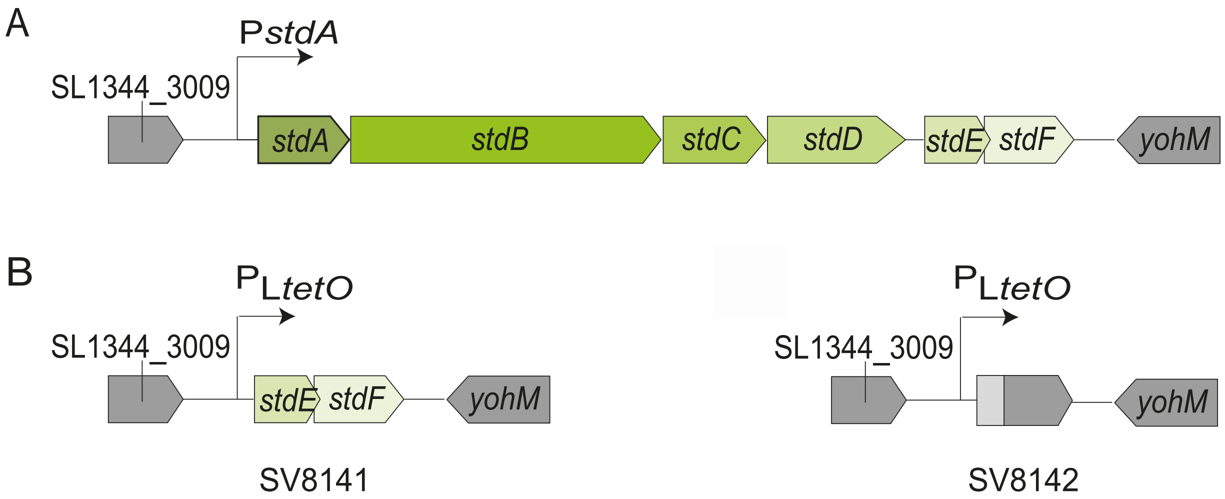

Figure S1. Diagrams of the *std* operon and of the *std* constructions carried by strains SV8141 and SV8142. **A.** The *std* operon and flanking loci in a *Salmonella enterica* wild type strain. Transcription of the operon is driven by the *stdA* promoter (P_{stdA}). **B.** Diagrams of the *std* operon and flanking genes in the strains used for transcriptomic analysis. SV8141 (P_{LtetO}-*stdEF*) and SV8142 (P_{LtetO}-Δ*stdEF*).
