# Supplementary figures and images for: Formation of phenotypic lineages in Salmonella enterica by a pleiotropic fimbrial switch
Source: PLoS Genet. 2018 Sep 25;14(9):e1007677. doi: 10.1371/journal.pgen.1007677 (PMC6173445; doi:10.1371/journal.pgen.1007677)

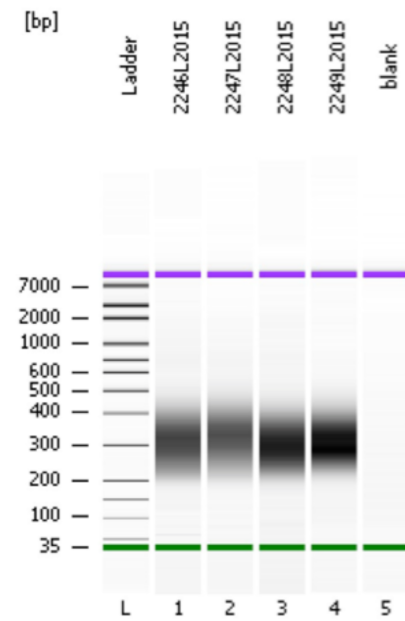

**Figure S2.** Electrophoresis file run summary of ChIP-seq samples.

Supplement: S2 Fig — (PDF) [file pgen.1007677.s006.pdf]
