# Supplementary material for: Formation of phenotypic lineages in Salmonella enterica by a pleiotropic fimbrial switch
Source: PLoS Genet. 2018 Sep 25;14(9):e1007677. doi: 10.1371/journal.pgen.1007677 (PMC6173445; doi:10.1371/journal.pgen.1007677)
Supplement: S3 Fig — (PDF) [file pgen.1007677.s007.pdf]

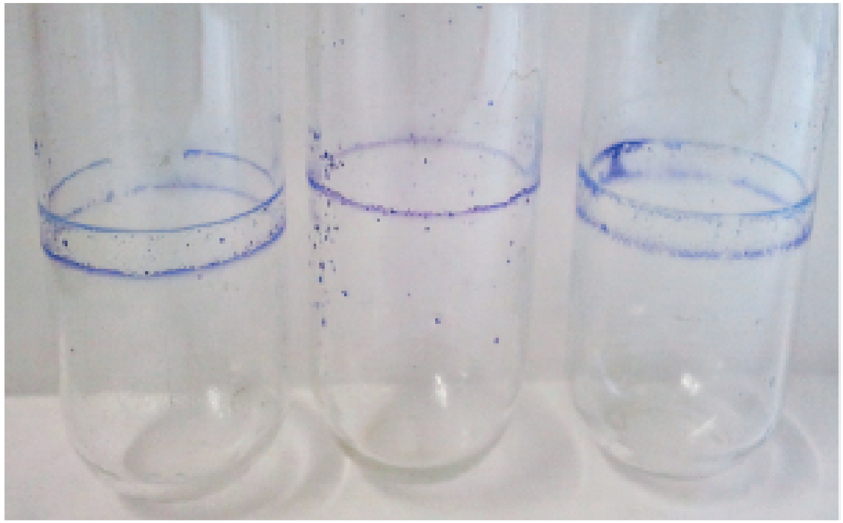

wt p_{LtetO}*stdEF* $p_{LtetO}\Delta$*stdEF*

Figure S3. Biofilm formation by the wild type strain. by SV8141 (P_{LtetO}-*stdEF*). and by SV8142 (P_{LtetO}-Δ*stdEF*). The biofilms were stained with crystal violet. The experiments were performed in triplicate. and a representative assay is shown.
